# Use of Solid-State NMR Spectroscopy for the Characterization of Molecular Structure and Dynamics in Solid Polymer and Hybrid Electrolytes

**DOI:** 10.3390/polym13081207

**Published:** 2021-04-08

**Authors:** Gabrielle Foran, Nina Verdier, David Lepage, Cédric Malveau, Nicolas Dupré, Mickaël Dollé

**Affiliations:** 1 Département of Chemistry, Université de Montréal, 1375 Avenue Thérèse-Lavoie-Roux, Montréal, QC H2V 0B3, Canada; nina.verdier@umontreal.ca (N.V.); david.lepage.3@cnrs-imn.fr (D.L.); cedric.malveau@umontreal.ca (C.M.); 2 Université de Nantes, CNRS, Institut des Matériaux Jean Rouxel, IMN, F-44000 Nantes, France; Nicolas.Dupre@cnrs-imn.fr

**Keywords:** solid-state NMR spectroscopy, polymer electrolytes, hybrid electrolytes, ion dynamics, electrochemistry

## Abstract

Solid-state NMR spectroscopy is an established experimental technique which is used for the characterization of structural and dynamic properties of materials in their native state. Many types of solid-state NMR experiments have been used to characterize both lithium-based and sodium-based solid polymer and polymer–ceramic hybrid electrolyte materials. This review describes several solid-state NMR experiments that are commonly employed in the analysis of these systems: pulse field gradient NMR, electrophoretic NMR, variable temperature T_1_ relaxation, T_2_ relaxation and linewidth analysis, exchange spectroscopy, cross polarization, Rotational Echo Double Resonance, and isotope enrichment. In this review, each technique is introduced with a short description of the pulse sequence, and examples of experiments that have been performed in real solid-state polymer and/or hybrid electrolyte systems are provided. The results and conclusions of these experiments are discussed to inform readers of the strengths and weaknesses of each technique when applied to polymer and hybrid electrolyte systems. It is anticipated that this review may be used to aid in the selection of solid-state NMR experiments for the analysis of these systems.

## 1. Introduction

Solid-state electrolytes for use in lithium batteries have been extensively studied as potential replacements to commercialized liquid electrolyte systems and have been touted as safer options as a result of being non-flammable, non-corrosive, and non-volatile [1,2]. There are three main classes of solid-state electrolytes: ceramic, polymer, and ceramic-polymer hybrids [3]. Depending on their crystal structure and their composition, ceramic electrolytes tend to have good lithium ion conductivity (generally on the order of 10^−4^ S/cm at ambient temperature which is about one to two orders of magnitude lower than liquid electrolytes however, Kato et al. have prepared Li_9.54_Si_1.74_P_1.44_S_11.7_Cl_0.3_ electrolytes with conductivities that rival those of liquid electrolytes) [4,5,6,7,8], good thermal stability, and adequate mechanical strength to impede dendrite formation [9,10]. However, these materials are rigid and tend to cause significant interfacial resistance when in contact with solid electrodes [10]. Polymer electrolytes tend to have good mechanical stability, minimal interfacial resistance with solid electrolytes, and are suitable for use in flexible battery applications [10]. However, lithium ion conductivity tends to be much lower in most polymer systems at room temperature than in liquid or some ceramic electrolytes (on the order of 10^−8^ to 10^−6^ S/cm at room temperature with lithium transference numbers below 0.5) and dendrite formation may still occur [11]. Hybrid electrolytes, which contain both solid polymers and ceramic particles, were created to capitalize on the advantages of both of these systems: they have higher lithium conductivities (on the order of 10^−5^ to 10^−4^ S/cm at ambient temperature) [12,13,14,15] than polymer electrolytes and are more flexible and interface compatible than ceramic electrolytes [16]. The ceramic particles can be either active or inert, where active ceramics are lithium conductors themselves and inert ceramics are non-conductive and are present to decrease crystallinity in the polymer electrolyte. However, lithium conductivities tend to remain lower than those of ceramic electrolytes as lithium ions must cross a complex series of polymer–ceramic interfaces for macroscopic-scale conductivity to occur [16]. In addition to complexities due to the presence of different electrolyte materials and the interfaces between them, microscale properties of the polymer electrolyte such as porosity and pore radii, along with the salt concentration and the possibility of concentration gradients, can impact ionic conductivity in polymer and hybrid electrolytes [17,18]. These complexities can make hybrid systems difficult to characterize. In addition to lithium-based all-solid-state batteries, similar systems have been developed using sodium as the mobile species. Solid-state sodium batteries, which have been in development since the 1980s, have been touted as being a more environmentally friendly technology relative to lithium-based batteries as a result of the higher abundancy and lower cost of sodium versus lithium [19,20]. Sodium polymer electrolytes tend to have ion conductivities on the order of 10^−7^ to 10^−6^ S/cm at ambient temperature whereas sodium hybrid electrolytes tend to have conductivities between 10^−6^ to 10^−5^ S/cm at the same temperature [21,22,23,24,25]. Due to a growing interest in improving materials for flexible battery systems, this review will focus on the characterization of molecular structure and ion mobility in polymer and hybrid electrolyte systems. Many characterization techniques have been employed in the analysis of these materials with some of the most frequently used being impedance spectroscopy, cyclic voltammetry, linear sweep voltammetry, differential scanning calorimetry, thermal gravimetric analysis, Raman spectroscopy, infrared spectroscopy, microscopy techniques, x-ray diffraction, and both solution-state and solid-state nuclear magnetic resonance (NMR) spectroscopy [26,27,28]. This review will focus on the use of solid-state NMR spectroscopy to analyze molecular structure and dynamics in polymer and hybrid electrolytes. NMR spectroscopy can be performed on any nucleus where spin, a quantized nuclear property, is non-zero [29]. Therefore, in addition to being non-destructive, NMR spectroscopy is an isotopically sensitive method for investigating several nuclei that typically exist in solid electrolytes [28]. For example, nuclei such as ^6^Li, ^7^Li, ^19^F, and ^23^Na are generally used to study dynamic processes whereas ^1^H and ^13^C tend to be used to characterize polymer structure [30,31]. NMR is particularly suited to this task as it is sensitive to changes in local environments that occur on a microsecond to second timescale [30]. In addition to being sensitive to small changes in chemical environments, solid-state NMR has the added advantage of allowing materials in solid electrolyte systems to be analyzed in their native state.

The purpose of this document is to introduce a series of solid-state NMR spectroscopy experiments that are commonly used in the analysis of solid polymer and hybrid electrolytes to an audience who studies these systems but may not have an extensive familiarity with solid-state NMR spectroscopy. To this end, each technique discussed in this manuscript will be introduced starting with the pulse sequence, a description of important setup parameters and specific advantages and disadvantages of applying the technique to lithium- and sodium-based systems. Additionally, readers will be directed to Table S1 (supplementary information) where information on required equipment and experimental timescales are provided. Where applicable, comparisons between the NMR technique and common electrochemical experiments will be provided. The purpose of these comparisons is to inform the reader of differences in information that can be determined via NMR and electrochemical methods as well as the advantages and disadvantages that are associated with each technique. Each section also includes examples of previous uses of the NMR experiment in the analysis of real solid-state polymer or hybrid electrolyte systems with the goal of demonstrating the use of these techniques in real systems. The significance of these works along with the findings and conclusions of their authors are presented. Although similar reviews on crystalline electrolytes and reviews focusing on specific polymers have been recently published, a comprehensive review on the use of solid-state NMR spectroscopy for the analysis of polymer electrolytes has not been produced in the past decade [32,33,34]. This work hopes to showcase the use of NMR spectroscopy in the analysis of recent electrolyte systems and to provide a more thorough description of the experiments themselves relative to what has been presented in previous work.

## 2. Solid State NMR Spectroscopy Techniques

### 2.1. Transference Number Determination

#### 2.1.1. Pulse Field Gradient NMR

Pulse field gradient (PFG) NMR is an experiment that is used to measure the self-diffusion coefficient of molecules or ions in solids or solutions where self-diffusion is the random translational motion that is driven by internal kinetic energy [35]. These motions typically occur on a local scale and are therefore not necessarily equivalent to the transference numbers, that can be directly measured via electrochemical methods, which describe ion motion on a macroscopic scale [15,36]. Additionally, the NMR technique cannot distinguish single ions from poorly dissociated ion pairs and clusters which tends to result in an underestimation of the self-diffusion coefficient [36,37,38].

**Figure 1:**
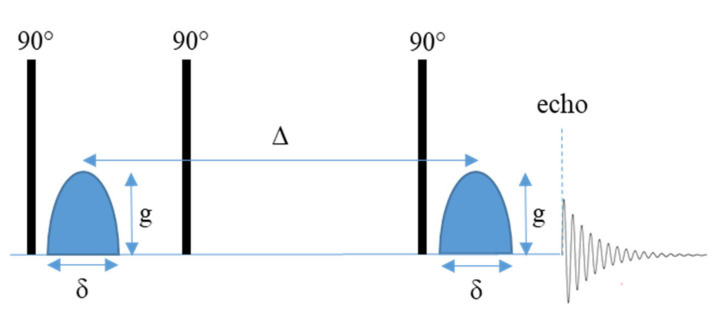
Stimulated echo pulse field gradient (PFG) pulse sequence.

PFG NMR experiments have previously been performed using a modified spin echo pulse sequence (90° pulse–180° pulse) where homogenous gradient pulses with duration δ and strength *g* are applied on either side of the 180° pulse [39]. However, the experiment is more commonly performed using a stimulated echo pulse sequence (Table S1). This experiment is generally considered to be more efficient, as the magnetization lies mainly along the z axis during the diffusion time (Δ) and is therefore subject to spin-lattice relaxation time (T_1_), instead of spin-spin relaxation time (T_2_) as in the spin-echo experiment. T_2_ is usually significantly shorter than T_1_ which is especially true in inhomogeneous systems. A drawback of the stimulated pulse sequence is the loss of half the signal, compared to the spin-echo experiment. The stimulated echo pulse sequence therefore allows the diffusion time to be longer [40]. The basic stimulated echo PFG pulse sequence is comprised of three 90° pulses (Figure 1) [40,41]. The application of the first 90° pulse initiates the preparation phase of the experiment where the first gradient pulse (with duration *δ* and magnitude *g*) is applied resulting in the Larmor frequency becoming spatially labeled in the direction of the gradient [35,39,40]. The gradient pulse is followed by the central pulse that causes the effective gradient to be zero as only the z component of the magnetization vector is of interest [39,40]. During this store phase spins are allowed to diffuse for a time (Δ) which is usually dependent on the spin-lattice relaxation of the system [39,40]. Due to the effective gradient being zero, the read and preparation phases are equivalent in terms of magnetization [40]. Therefore, the application of the second gradient pulse, which occurs during the read phase after the application of a third 90° pulse, either cancels out the effects of the first gradient pulse if no motion has occurred or results in an attenuation of the total signal that is proportional to the translational motion that has taken place [35,39,40]. PFG NMR can be used to measure diffusion along any axis depending on how the x, y, and z gradients are used [35,42].

In the study of solid polymer or hybrid electrolytes, PFG NMR spectroscopy is commonly used to measure self-diffusion coefficients of anions and cations of the salt species as these are dissociated in the polymer or hybrid network. This is typically accomplished by performing ^7^Li (cation mobility) and ^19^F (anion mobility) experiments as both nuclei have high magnetic susceptibilities [43]. Additionally, PFG NMR can be performed on ^1^H and ^13^C to measure the mobility of the polymer chains as this motion can have a significant impact on the overall ion mobility in these systems [44,45,46]. PFG NMR is not usually used to measure sodium diffusion in sodium-conducting electrolytes because quadrupolar effects cause relaxation at ^23^Na sites to be much faster than relaxation at ^7^Li sites [47]. Transport numbers for sodium ions therefore tend to be measured via electrochemical methods such as potentiostatic polarization [48]. Larger self-diffusion coefficients generally indicate higher ion mobility. Self-diffusion coefficients (*D*) can be calculated based on the degree of signal attenuation for each peak in the NMR spectrum using the Stejskal-Tanner equation (Equation (1)) [39,42], where *I* and *I_0_* are the spectral integrals with and without the application of the gradient pulses, *γ* is the gyromagnetic ratio of the measured nucleus, *g* is the gradient strength, *δ* is the gradient pulse duration and Δ is the diffusion time. Usually, *D* is calculated by fitting the plot of signal intensity as a function of the gradient strength, keeping all delays and gradient duration constant. This allows the effects of relaxation on signal intensity to be removed. The Stejskal-Tanner equation is used for the spin echo experiment. When PFG NMR is performed using other pulse sequences, the Δ and *δ* quantities are modified to take the parameters of the experiment into account.

(1) $$I=I_{0}\exp\left(-\gamma^{2}g^{2}\delta^{2}D\left(\Delta -\frac{\delta }{3}\right)\right)\;\;\;\;\;\;$$

Self-diffusion coefficients (*D_+_* the diffusion coefficient for the cation and *D_−_* the diffusion coefficient for the anion), which are used to gauge the mobility of ions in a system, can also be used to calculate the activation energy for local-scale ion motion by plotting these values in an Arrhenius plot [49].

Additionally, the transference number (*t_NMR_^+^*), which is a measure of cation mobility relative to anion mobility (mole of cation transferred by migration per Faraday of charge) in a dilute system with no concentration gradients or ion association, can be calculated using self-diffusion coefficients (Equation (2)) where *z_+_* and *z_−_* are the charges on the cation and anion respectively [50,51]. This method for measuring the transference number works best in dilute systems as it assumes negligible ion-ion interactions and does not take into account the motion of associated species (sees everything as ions) [51].

(2) $$t_{NMR}{}^{+}=\frac{z_{+}D_{+}}{z_{+}D_{+}-z_{-}D_{-}}\;\;\;\;\;\;\;$$

As mentioned previously, transference numbers can also be experimentally determined using electrochemical methods. A commonly used method is to couple a potentiostatic polarization with electrochemical impedance spectroscopy (EIS). This is typically done by employing the Bruce-Vincent method which involves polarizing a Li || Li cell using a small potential difference (~10 mV) until a constant current is reached [51,52]. The resultant steady state conditions can then be used to calculate the transference number (*t_PP_*) via Equation (3) where *ΔV* is the applied potential difference, *I_0_* is the initial current, *I_ss_* is the steady state current, *R_o_* is the resistance prior to polarization, and *R_ss_* is the resistance after polarization [51]. As for the NMR measurement, this approach is only valid in systems where the concentration gradient is negligible [51]. In more concentrated systems ([Li^+^]/[O] > 0.1), the potentiostatic polarization experiment results in the diffusion of neutral ion pairs resulting in the over estimation of *t_PP_* [51,53].

(3) $$t_{PP}=\frac{I_{ss}\left(\Delta V-I_{o}R_{o}\right)}{I_{0}\left(\Delta V-I_{ss}R_{ss}\right)}\;\;\;\;\;\;\;$$

To better represent ion diffusion in concentrated solutions, the Balsara-Newman method can be used [54]. The Balsara-Newman method for the determination of transference numbers is an extension of the Bruce-Vincent method that takes into account concentrated solution theory such that the contribution of ion pairs and clusters in bulk ionic motion can be accounted for [55]. With this approach, the transference number has a complex dependence on salt concentration and becomes negative when ion transport is dominated by ion clusters. While the Balsara-Newman method has the advantage of accurately measuring transference number in concentrated solutions, the method tends to be experimentally demanding as several parameters must be determined experimentally including the ionic conductivity (*σ*) from EIS, the steady-state transference number (*t_SS_*) from potentiostatic polarization, the restricted diffusion coefficient (*D*) from PFG NMR, and the potential of the concentrated cell (*U*). These parameters are combined with the bulk electrolyte concentration (*c*), the molality of the solution (*m*), ionic charge (*z*), the total number of ions when the salt dissociates (*v*), the number of cations when the salt dissociates (*v_+_*), and Faraday’s constant (*F*) to obtain the transference number (*t_Ne_*) (Equation (4)).

(4) $$t_{Ne}=1-\frac{{\left(v_{+}z_{+}\right)}^{2}}{v}\frac{2FDc}{\sigma }\left(1-\frac{1}{t_{SS}}\right)\left(\frac{d\ln m}{dU}\right)\;\;\;\;\;\;\;$$

The Hittorf method (also called Tubandt when applied to solids) is another electrochemical technique, suitable for use in concentrated solutions, that can be used in the experimental determination of transference numbers in solid polymer electrolytes [56]. The electrolyte is subdivided into sections where each fragment is placed between polarized electrodes and is subjected to polarization by a known charge [51,57]. The transference number of the anion can be determined based on the change in the number of moles of the cation following polarization of the compartment that is closest to the cathode as shown in Equation (5) where *Δm* is the change in the number of moles of cation, *F* is the Faraday constant, and *Q* is the amount of charge that was passed through the compartment.

(5) $$t_{+}=1+\frac{\Delta mF}{Q}\;\;\;\;\;\;\;$$

Bruce et al. used the Hittorf method to measure *t***_+_** in a poly(ethylene oxide) (PEO)-LiClO_4_ electrolyte [57]. *t_+_* was found to be 0.06 which is lower than what has been previously reported by PFG NMR and potentiostatic polarization: 0.2 to 0.3 [57]. The differences between the transference numbers can be attributed to the fact that the Hittorf method does not measure the mobility of neutral species because the charge is not applied for a long enough period for a concentration gradient to develop in each subsection of the polymer. Therefore, transference numbers can be overestimated if significant quantities of ion pairs are formed [57]. Although the Hittorf method specifically measures charged species, it can be difficult to perform with polymer electrolyte samples because PEO-based samples are often too adhesive to be easily subdivided into sections. Additionally, the technique requires a specialized electrochemical cell, and the sample must be divided into at least four different compartments, making it difficult to obtain accurate results [51].

Differences in transference numbers calculated via PFG NMR and various electrochemical techniques can be evaluated in the context of the dissociation ratio of the ionic species. The dissociation ratio describes the extent to which the ionic species are dissolved in a sample, with 1 indicating perfect dissociation and 0 indicating no dissociation [58]. As PFG NMR does not distinguish between dissociated and un-dissociated species, transference numbers reported via PFG NMR tend to be artificially higher than those determined via electrochemical methods as the motion of neutral pairs, dissociated ions, and charged clusters is usually included in the measurement [58,59]. It can therefore be concluded that in samples where similar transference numbers are achieved via NMR and electrochemical methods, nearly complete dissociation of the ionic species has been achieved.

**Figure 2:**
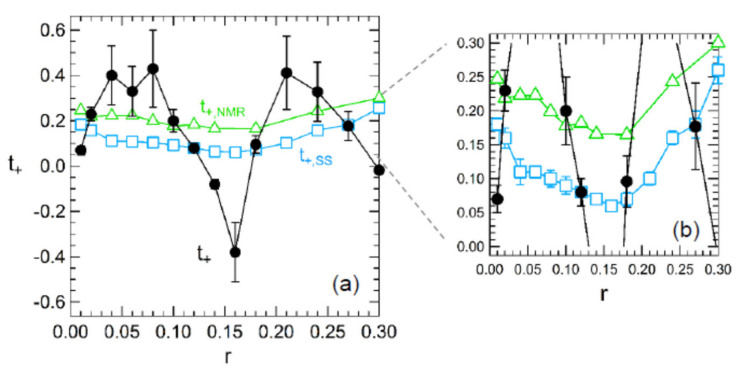
(**a**) Transference number for Li+ in a poly(ethylene oxide) (PEO)- lithium bis(trifluoromethanesulfonyl)imide (LiTFSI) system as a function of the molar ratio of lithium ion to ether oxygens (r) measured via PFG NMR (t+,NMR), potentiostatic polarization (t+,SS) and the Balsara-Newman method (t+). (**b**) Inset where y axis is set between 0 and 0.3 to emphasize differences in transference numbers determined via PFG NMR, potentiostatic polymerization and the Balsara-Newman method. © Journal of the Electrochemical Society, 2017 [53].

Several examples of experiments that illustrate these differences between PFG NMR and electrochemical methods are described here. For example, a study of lithium bis(trifluoromethanesulfonyl)imide (LiTFSI)-doped PEO, which is discussed here for the purpose of demonstrating that different transference numbers can be determined for the same system using different experimental methods, showed that transference numbers as a function of the molar ratio of lithium ion to ether oxygens (*r*) varied between 0.17 and 0.30 based on PFG NMR data, between 0.06 and 0.26 based on potentiostatic polarization experiments and between −0.38 and 0.45 by the Balsara and Newman method, from *r* = 0 to *r* = 0.3 with the lowest value of −0.38 being observed at *r* = 0.16 (Figure 2) [53,55]. The difference between the values calculated based on PFG NMR and potentiostatic polarization which depend on dilute solutions and those calculated based on the Balsara-Newman method suggest that the ionic species are not completely dissociated in this system.

However, transference numbers determined by electrochemical methods can also be subject to overestimation. This was demonstrated by Sun et al., who showed that potentiostatic polarization can cause the diffusion of neutral species in concentrated systems and can result in an over estimation of the transference number via electrochemical methods [36]. Li^+^ transference numbers in a poly(trimethylene carbonate)-ε-caprolactone copolymer were 0.32 via PFG NMR and 0.66 via potentiostatic polarization [36]. These differences were attributed to the migration of neutrally charged aggregates and could be avoided by using the Hittorf method [36]. Work by Tominaga and Yamazaki showed that the overestimation of transference numbers measured via PFG NMR and potentiostatic polarization can even differ within the same study [60]. Their comparison of transference numbers in PEO-lithium bis(fluorosulfonyl)imide (LiFSI) (0.11 via potentiostatic polarization and 0.24 via NMR) and in poly(ethylene carbonate) (PEC)-LiFSI (0.54 via potentiostatic polarization and 0.24 via NMR) showed that the degree of ion dissociation in their prepared materials was not consistent [60]. These differences demonstrate that understanding the factors that influence transference numbers measured via both techniques is important when interpreting data.

Despite limitations related to the extent of salt dissociation, previous PFG NMR studies have demonstrated that Li^+^ transference numbers can be dependent on both the segmental motion of polymer chains and the affinity of the lithium cation for the polymer. For example, Xu et al. and Sun et al. have found that cation mobility tends to be lower than anion mobility in LiTFSI-doped PEO at high lithium loading as the cation has increased affinity for the polymer matrix [27,36]. As a result, segmental motion of polymer chains can impact short- and long-range Li^+^ diffusion [36]. Therefore, PFG NMR experiments have been performed to demonstrate that interventions designed to increase segmental motion such as the synthesis of block co-polymers [61], the addition of inert ceramic fillers [60,62] and increased sample temperature [63,64] can increase the mobility of Li^+^ in polymer electrolytes. The above experiments are important as they provide insight into the fact that polymer segment mobility, in addition to the extent of ion dissociation, contributes to observed transference numbers.

**Figure 3:**
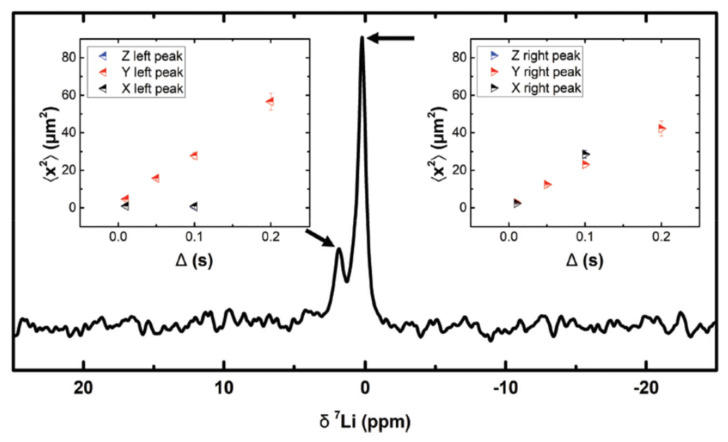
One dimensional slice from a ^7^Li PFG NMR (7.1 T) experiment of lithiated liquid electrolyte in a porous silicon wafer. The inserts show calculated mean square displacements for each lithiated environment as a function of diffusion time. Reproduced from ref. [42] with permission from the PCCP Owner Societies.

The above examples have focused on free ion diffusion: the random diffusion of ions that occurs as a result of thermal energy. However, PFG NMR can also be used to measure confined diffusion where the motion of ions is impeded by encountering boundaries [42]. The possibility to measure confined diffusion with three dimensional PFG NMR (using x, y and z gradients) may allow for more precise mapping of lithium-ion diffusion in electrolyte systems [42]. Engelke et al. performed ^7^Li PFG NMR to follow the three-dimensional motion of a lithiated liquid electrolyte in a porous silicon wafer to map the pore structure of the wafer (Figure 3) [42]. The dimensions of the pores in the wafer were determined by calculating the mean square displacement of the lithium ions using their self-diffusion coefficients [42]. Although Engelke et al. performed their experiments on a confined liquid, similar methodology could be applied to polymer or hybrid electrolytes.

**Figure 4:**
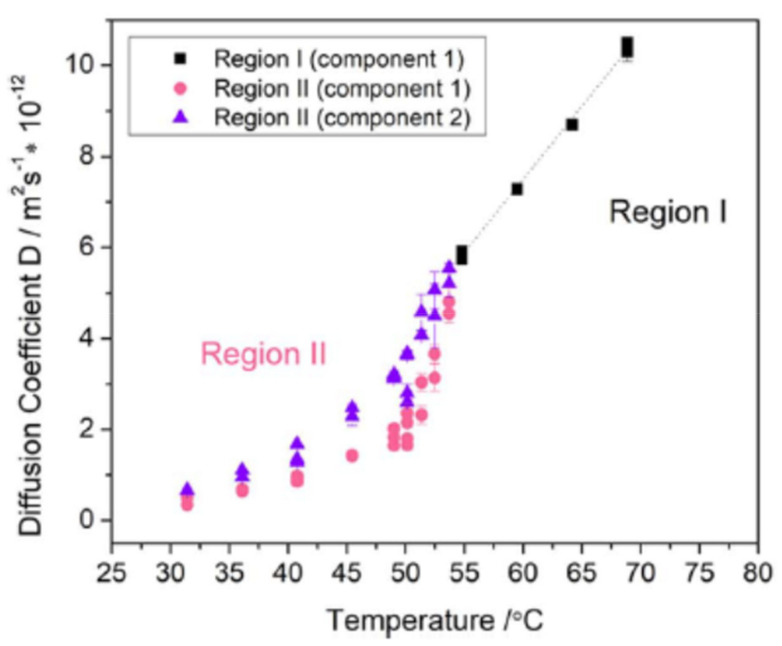
Diffusion coefficients from the hybrid ceramic-polymer electrolyte as a function of temperature as determined by PFG NMR. © Journal of the Electrochemical Society, 2020 [65].

The three-dimensional PFG NMR experiment could be particularly useful in the study of hybrid polymer electrolytes that contain inert ceramics as it could be used to determine the position of these particles and ascertain how Li^+^ ions pass through the electrolyte. The analysis of electrolytes containing active ceramics is more complex as there is the potential for multiple mobile lithium species resulting in multiple diffusion coefficients. To demonstrate the use of PFG NMR in the analysis of a hybrid electrolyte, a system containing 70% PEO, 12% LiTFSI, 9% Li_0.34_La_0.51_TiO_3_, and 9% succinonitrile (all weight percent) was analyzed. PFG NMR showed the existence of two mobile lithium species between 30 and 55 °C and a single mobile lithium species between 55 and 70 °C (Figure 4) [65]. Although a single peak was observed via 1D ^7^Li NMR, the diffusion curve was best fit using two components between 30 and 55 °C [65].

These diffusion coefficients were attributed to lithium conduction in the polymer and lithium conduction in the active ceramic. As the transition from two mobile lithium species to one mobile lithium species occurs around the melting point of PEO, it has been proposed that the associated decrease in polymer crystallinity results in lithium conduction occurring preferentially through the polymer at high temperatures [65]. At lower temperatures, where the polymer is expected to be crystalline, the presence of two diffusion coefficients suggests that both the polymer and ceramic participate in lithium conduction [65]. This example illustrates the primary advantage of PFG NMR over electrochemical techniques: the ability to measure species-specific diffusion coefficients in multi-component systems. This is particularly welcome in the analysis of hybrid electrolytes containing active ceramic fillers as both components of the electrolyte may participate in lithium transport. However, two-dimensional exchange spectroscopy experiments (described in Section 2.3) would be needed to quantify ion exchange between these components.

#### 2.1.2. Electrophoretic NMR

**Figure 5:**
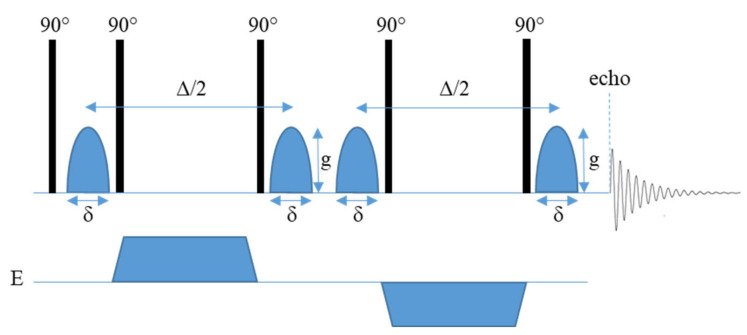
Electrophoretic NMR pulse sequence.

Electrophoretic NMR is a one-dimensional diffusion method, that is used to measure the flow of ions, from which transference numbers can be derived [66,67,68]. This experimental method, involving a double stimulated echo and an applied electric field, has been previously used for the characterization of ion mobility in liquid electrolytes [66,69]. However, it has had limited application in solid-state electrolytes outside of the work of Rosenwinkle and Schönhoff who used the method to determine transference numbers for lithium cation motion in PEO doped with various concentrations of LiTFSI [69]. The method is similar to PFG NMR in the sense that the pulse sequence is comprised of a series of gradient pulses with length *δ* and strength *g* within an echo comprised of 90° pulses (Table S1) (Figure 5). In the absence of the electric field, the random translational motion of mobile species in the direction of the magnetic field gradient results in random phase shifts and thus loss of net magnetic phase coherence, as in standard NMR diffusion experiments [66]. However, because the polarization of the electric field is switched after the first half of the pulse sequence to cancel out non-electrophoretic directional motion, only directional motion contributes to the measured phase shift (*ø*) [69].

The electrophoretic drift of the observed species is signified by a phase shift (*ø*) in the NMR signal when the gradient strength (*g*) is changed. The phase shift can be calculated using Equation (6) where *γ* is the gyromagnetic ratio of the observed nuclei, *δ* is the gradient pulse length, *g* is the gradient strength, Δ is the observation time, *E* is the strength of the applied electric field, and *μ* is the electrophoretic mobility which is used to calculate the transference number.

(6) ø=γδgΔEμ       

**Figure 6:**
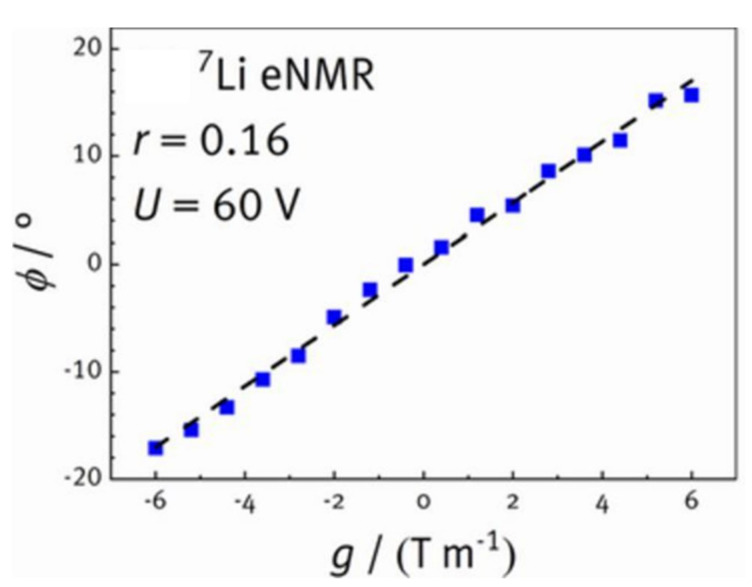
Plot of phase shift, determined via electrophoretic NMR, as a function of gradient strength for lithium cations in LiTFSI-doped PEO at 90 °C and a molar ratio of lithium to ether oxygen of 0.16. © Journal of the Electrochemical Society, 20 [69].

The electrophoretic mobility, which can be extracted from a plot of phase shift as a function of electric field strength using equation (6) is proportional to the drift velocity (*v*) and the electric field strength (Equation (7)). The drift velocity for free cations is defined as positive while the drift velocity for free anions is defined as negative. This relationship remains true when a plot of the phase shift as a function of gradient strength remains linear (Figure 6).

(7) v=μE       

While the electrophoretic mobility can be impacted by electric field-induced changes in polymer chain orientation and electroosmotic drift, these effects can be canceled by extrapolating the phase shift as a function of field strength to zero electric field [69]. The electrophoretic mobilities of the cation and the anion (*μ_+_* and *μ_−_*) can be used directly to calculate transference number (*t_eNMR_*) using Equation (8).

(8) $$t_{eNMR}=\frac{\mu_{+}}{\mu_{+}-\mu_{-}}\;\;\;\;\;\;$$

A PEO-LiTFSI system was used to demonstrate that transference numbers in solid systems can be determined via electrophoretic NMR. These experiments showed that cation mobility decreased with increasing salt content as a result of coordinating interactions between the salt and the polymer causing decreased side chain mobility [69]. The authors compared the *t_eNMR_* values with transference numbers obtained via PFG NMR (*t_PFG_*). Transference numbers obtained via both techniques were found to be in close agreement (*t_PFG_*: 0.21, 0.17, 0.17 and *t_eNMR_*: 0.23, 0.19, 0.15) for lithium at ether oxygen ratios of 0.06, 0.10, and 0.16 respectively, indicating that the samples contained mostly free cations [69]. As electrophoretic mobility is less impacted by the presence of ion pairs and agglomerations, it is anticipated that it can be used to provide more accurate values for transference numbers than PFG NMR in undiluted media. A direct comparison between electrophoretic NMR and electrochemical methods would be needed to determine the relative strengths of these methods for the determination of transference numbers in various types of samples. However, a lesser reliance on dilute systems suggests that electrophoretic NMR would be particularly useful in the analysis of systems with multiple mobile lithium environments, such as hybrid electrolytes containing active ceramics.

### 2.2. Variable Temperature NMR

As lithium and sodium conductivities are temperature-dependent in many solid polymer and hybrid electrolytes, variable temperature NMR spectroscopy has been used to evaluate ion dynamics in these systems. Experiments that have been regularly performed for site-specific analysis of molecular dynamics include spin-lattice (T_1_) relaxation, spin-spin (T_2_) relaxation and linewidth analysis. Pulse sequences used to perform these techniques, along with examples of their use in the study of solid-state polymer and hybrid electrolytes, are discussed in this section. It must be noted that the larger quadrupolar moment of sodium, relative to lithium, means that quadrupolar coupling may have a significant impact on these parameters.

#### 2.2.1. Spin Lattice Relaxation (T_1_)

**Figure 7:**
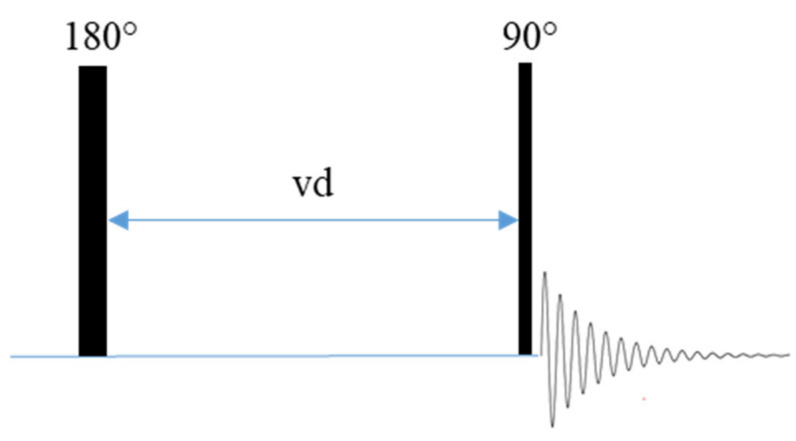
Inversion recovery pulse sequence.

The application of radio frequency pulses during NMR experiments perturbs the magnetization vector away from its equilibrium position. The process through which the magnetization vector returns to equilibrium is termed relaxation [29]. In this work, the impact of temperature on T_1_ relaxation for the study of ion mobility in solid electrolyte systems will be discussed. T_1_ relaxation is characterized by the flow of energy out of the spin system and into the lattice and can be conceptualized as the time required for a perturbed magnetization vector to return to its equilibrium position following the application of a radio frequency pulse [29,70]. T_1_ relaxation generally occurs on the order of seconds to milliseconds but can be up to several minutes long for some nuclei in some environments. This relaxation process can be influenced by properties of the sample such as ion mobility and dipolar and quadrupolar interactions [70]. T_1_ relaxation is typically measured using the inversion recovery pulse sequence (Table S1) (Figure 7) which is comprised of a 180° pulse which inverts all signals, a variable delay time (vd) during which relaxation back to equilibrium is allowed to occur, and a 90° pulse for spectral acquisition [29].

This experiment produces a series of spectra of differing signal intensity as a function of variable delay time. *T*_1_ relaxation can be calculated by fitting a plot of signal intensity as a function of variable delay time (*vd*) with one (or a series of) exponential functions. The fit can be performed using Equation (9) where *S_vd_* is the signal intensity at a given variable delay time and *S*_0_ is the signal when *vd* is equal to zero [70,71]. *K* is a fitted constant (with a value that is close to but does not exceed 2) that accounts for imperfect 180° pulses and the use of non-optimal repetition times.

(9) $$S_{vd}=S_{0}\left(1-Ke^{\frac{-vd}{T_{1}}}\;\right)\;\;\;\;\;$$

It is worth noting that as a quadrupolar nuclei (spin greater than ½), *T*_1_ build up curves for ^7^Li spectra should in theory be fit using a double exponential function [72,73]. However, as a result of the small quadrupolar moment of ^7^Li (~0.04 MHz) [74], deviations from a single exponential fit are difficult to detect [72]. The *T*_1_ relaxation time can also be quickly approximated using the variable delay time that results in net zero magnetization (*T_null_*) (Equation (10)) [70].

(10) $$T_{1}=\frac{T_{null}}{\ln2}\;\;\;\;\;\;\;$$

Meanwhile, ^23^Na has a much larger quadrupolar moment which ranges between close to 0 and up to 7 MHz depending on the symmetry of the internal coordination sphere with highly symmetrical coordination spheres giving rise to low quadrupolar moments and coordination spheres with low symmetry giving rise to larger quadrupolar moments [75]. Therefore, *T*_1_ build-up curves coming from sodium-based electrolytes may need to be fit using two or more exponentials depending on the magnitude of the quadrupolar moment.

NMR spectroscopy is highly sensitive to the impact of polymer chain dynamics on T_1_ relaxation times [72]. As cation mobility and the mobility of the polymer backbone generally occur on the order of microseconds to seconds, these quantities can be evaluated via T_1_ relaxation [76]. Many researchers have measured T_1_ relaxation as a function of temperature to study the impact that this variable has on Li^+^ and polymer chain segmental mobility [76,77]. As activation energy for these processes can be calculated based on Arrhenius and/or Vogel-Tamman-Fulcher (VTF) plots of T_1_ values, these quantities can be directly coupled with EIS data to provide a site-specific view of which motional processes contribute to long-range lithium conductivity [72,78].

When calculating activation energies from T_1_ values, temperature-dependent changes in ionic conductivity in polymer electrolytes must be taken into account as a result of changes that occur in polymer chain mobility above and below the glass transition temperature (T_g_) [78]. At low temperatures, ionic conductivity in polymers is typically a result of the dissociated ionic pairs migrating through interstitial defects [78]. The activation energy for this process can be described by the Arrhenius behavior. Above T_g_, ionic transport increases as a result of increased motion in the polymer side chains as they become less crystalline [79]. The activation energy for this process is best described by the VTF equation which was originally developed to describe the viscosity of supercooled liquid (Equation (11)) [78], where *σ* is the ionic conductivity, *A* is a pre-exponential factor that is related to the concentration of the charge carrier, *E_a_* is the activation energy which is typically related to the segmental motion of the polymer chains, *R* is the ideal gas constant, *T_o_* is the Vogel temperature which is equal to the glass transition temperature in ideal glasses [80]. This is typically taken to be 50 °C below the glass transition temperature in polymer electrolytes [80].

(11) $$\sigma =Ae^{\frac{E_{a}}{R\left(T-T_{o}\right)}}\;\;\;\;\;\;\;$$

Lower T_1_ values are typically associated with increased mobility in solids. This is because spins that require changes in energy to relax can transfer that energy to or from motional processes [81]. An example that illustrates the impact motional processes on the rate of T_1_ relaxation can be found in the work of Peng et al. who compared ^7^Li T_1_ relaxation times of a Li_2_O-Al_2_O_3_-SiO_2_-P_2_O_5_-TiO_2_-GeO_2_ (LICGC) ceramic, a mobile lithium-containing polymer phase and an immobile lithium-containing polymer phase in a PEO-LiTf-LICGC hybrid electrolyte [82]. T_1_ relaxation times were longer for all lithium-containing phases of the hybrid electrolyte relative to the plain LICGC sample and a PEO-LiTf electrolyte [82]. The observed lower lithium-ion mobility was attributed to a Lewis acid/based interaction between the polymer and the ceramic creating increased resistance in the ceramic-polymer interfacial layer [82]. Other interactions that can increase the rate of T_1_ relaxation include paramagnetism and dipolar or quadrupolar coupling. Paramagnetic samples contain unpaired electrons which have large magnetic moments and are particularly effective at promoting relaxation [81]. Paramagnetism is particularly relevant in hybrid systems as many ceramics contain paramagnetic transition metals. Dipolar and/or quadrupolar coupling interactions tend to decrease T_1_ relaxation times by providing an alternate pathway through which the relaxation process can occur [81]. This means that coupling interactions between ions and the membrane can also influence T_1_ relaxation with stronger interactions resulting in decreasing T_1_. However, as lower T_1_ values are typically associated with more mobile as opposed to membrane-bound Li^+^ ions, it can be assumed that motional processes have a more significant impact on T_1_ relaxation in these systems than dipolar coupling interactions do.

Similar relationships between T_1_ relaxation and site mobility have been observed in sodium-based polymer electrolytes. Schantz and Kakihana observed two sodium sites in a poly(propylene oxide) sample that contained NaCF_3_SO_3_ [83]. The observation of two distinct sites (one narrow and one broad) was attributed to the presence of two different nuclear coordination environments which were made visible by differences in quadrupolar coupling between these sites [75,83]. The narrow site, which had a T_1_ relaxation time of 9 μs, was attributed to mobile dissociated ions whereas the broad site (with a T_1_ relaxation time of 13 ms) was attributed to ion pairs which tend to have low mobility [83]. Similar observations regarding the impact of motional processes on T_1_ relaxation in sodium-based polymer systems were made by Pak et al. in a poly(propylene oxide)-NaB(C_6_H_5_)_4_ system [84]. This sample also contained two sodium sites which could be attributed to mobile dissociated ions and less mobile ion pairs [84]. The T_1_ relaxation time for the mobile site was about 10 ms at room temperature with the T_1_ relaxation time for the broad site being about ten times longer. The difference between these relaxation times, and the faster T_1_ relaxations times that are found in most sodium-containing polymer electrolytes such as those studied by Schantz and Kakihana, was stated to be a result of complexation between the sodium cation the large B(C_6_H_5_)_4_ anion [83,84]. It was additionally observed that the T_1_ relaxation continued to decrease with increasing temperature and did not plateau at the T_g_ of poly(propylene oxide) as has been previously observed in several similar systems [84]. This suggests that ion motion primarily occurs independently of the motion of the polymer chain.

**Figure 8:**
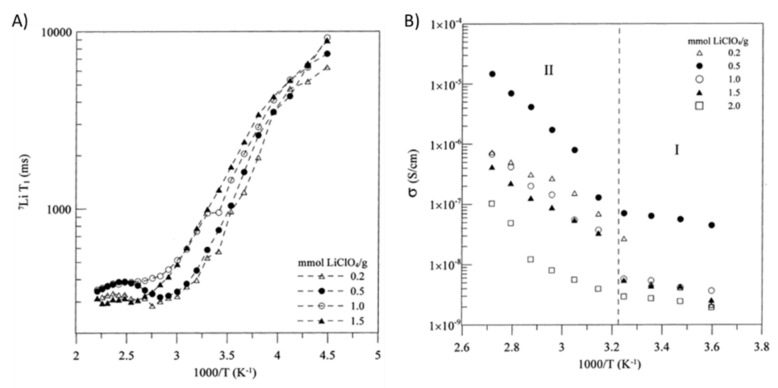
(**A**) Variable temperature T_1_ relaxation times for a series of LiClO_4_-doped polyurethane-poly(dimethylsiloxane) copolymers with various salt concentrations. (**B**) Ionic conductivity as a function of temperature for a series of LiClO_4_-doped polyurethane-poly(dimethylsiloxane) copolymers with various salt concentrations. Reprinted with permission from [76]. Copyright 2002 American Chemical Society.

Sample mobilities can also be compared using a quantity called T_1_ minimum. The T_1_ minimum occurs at the temperature where the variations in relaxation rate are on the order of the Larmor frequency [30,76]. The T_1_ minimum can more directly be described as the temperature at which T_1_ relaxation occurs most rapidly [81]. Species where the T_1_ minimum occurs at a higher temperature have lower ion mobility as more energy must be transferred to or from the spin system for the maximum rate of T_1_ relaxation to occur [30,76,81]. The correlation between differences in T_1_ minimum and differences in ionic mobility was demonstrated through the analysis of LiClO_4_ doped polyurethane-poly(dimethylsiloxane) copolymers [76]. In this system, the temperature where the T_1_ minimum occurred was found to increase with increasing LiClO_4_ loading (Figure 8A) [76]. These findings were well-correlated with impedance spectroscopy data as samples with T_1_ minima at higher temperatures were found to be less conductive (Figure 8B) [76]. Although the determination of the T_1_ minimum is generally time consuming and requires a probe and a variable temperature control with an extensive range, it can provide more information on the thermal properties of the polymer system in question than more typical T_1_ measurements, allowing for potential comparisons to differential scanning calorimetry data and the analysis of materials or samples that are not amenable to differential scanning calorimetry.

In the example presented in Figure 8, activation energies were calculated based on the low temperature T_1_ data by fitting the slope of an Arrhenius plot [76]. The activation energy was found to increase from 6.3 to 11.3 kJ/mol when the molar ratio between the lithium salt and the polyurethane-poly(dimethylsiloxane) copolymer was increased from 0.2 to 1.5 [76]. Activation energies were not calculated for the high temperature region as a result of a kink in the T_1_ data around 0 °C corresponding to the transition from Arrhenius to VTF conductive behavior in the polymer sample, thus indicating a link between NMR and conductivity measurements [76].

Lin et al. and Daigle et al. have measured variable temperature T_1_ in polyethylene glycol (PEG)-based co-polymer systems containing lithium salts [31,76]. In both cases T_1_ was observed to decrease with increasing temperature indicating increased mobility of both the polymer segment and the Li^+^ ions. These results can often be correlated with conductivity data. In these works, the low temperature portion of the T_1_ buildup as a function of temperature curve is used to calculate activation energies for Li^+^ transport in these systems, a method that can be used in samples that are not suitable for analysis via EIS [31]. It is worth noting that this method does present some challenges as it requires the ability to measure NMR spectra at low temperatures (down to −50 °C in Lin et al. [76]) since temperatures above about −10 °C are around the T_1_ minimum where significant changes in T_1_ as a function of temperature no longer occur [31]. An additional challenge is peak overlap. Despite differences in T_1_ relaxation times between sites, Lin et al. reported that it was difficult to deconvolute signals from the polymer backbone and the mobile Li^+^ species, resulting in the reported activation energies potentially including contributions from both species.

The differences between characterizing Li^+^ mobility in a polymer electrolyte using T_1_ relaxation and EIS are illustrated in the outcome of the study by Jeon and Kwak [72]. The variable temperature T_1_ analysis of a poly(vinylidene fluoride-co-hexafluoropropylene) network doped with P(EO-EC)-LiCF_3_SO_3_ between −40 and 70 °C showed that T_1_ relaxation time and T_1_ derived activation energy for lithium motion decreased with increasing temperature and increasing P(EO-EC)-LiCF_3_SO_3_ doping [72]. Both are consistent with increased lithium mobility. Decreasing activation energy was also observed with increasing temperature and increasing P(EO-EC)-LiCF_3_SO_3_ doping via EIS [72]. However, the activation energies were not equal. The activation energy that was calculated based on T_1_ relaxation (9.8–13.1 kJmol^−1^) was lower than that which was calculated based on lithium conductivity (41.2–50.1 kJmol^−1^) [72].

The difference in activation energies is a result of NMR and EIS measuring ion motion on different length scales. NMR effectively measures local-scale mobility while EIS measures global mobility. It is anticipated that the higher activation energy as measured by EIS is a result of more energy being required for long-range motion than for local-scale motion [72]. Additionally, in samples with multiple lithiated chemical environments, site-specific activation energies, which may not contribute to global ionic conductivity, can be determined by NMR. This is not the case with impedance spectroscopy of polymer samples as one conductivity value typically represents all components of a sample that are in the same phase and that contribute to long-range ionic conductivity. There may however be the possibility of distinguishing multiple phases via EIS in ceramic or hybrid samples due to the presence of multiple semi-circles. The difference in motional scale, along with the possibility to measure site-specific mobility in NMR are the reasons why activation energies calculated via these techniques may not match and should not be compared directly. However, comparing these properties qualitatively (increasing or decreasing) can be done to support claims of increased or decreased mobility in a sample.

#### 2.2.2. Spin-Spin Relaxation (T_2_)

T_2_ relaxation, also called transverse relaxation or spin-spin relaxation, is the relaxation of the x and y components of the magnetization vector without energy transfer to the lattice [70]. This process occurs as the magnetization vector precesses about the xy plane after a pulse has been applied [70]. During this time, individual spins that make up the magnetization vector fan out across the xy plane which destroys coherence (the orientation of magnetization in the same direction) and can result in line broadening. This process is due to small fluctuations of local microscopic magnetic field caused by local mobility. This effect, which depends on the orientation of the nucleus with respect to the magnetic field and the scalar coupling in electrons is particularly apparent when T_2_ relaxation times are short [70]. Short T_2_ relaxation times can cause significant signal loss if the T_2_ is shorter than the delay times that are used in some pulse sequences. T_2_ relaxation times can be particularly affected by the quadrupolar interaction as it is also orientation dependent and heavily dependent on the structure of the local coordination sphere [85]. Changes in the quadrupolar interaction tend to minimize T_2_ relaxation time which causes significant line broadening [85]. Line broadening can complicate spectral interpretation by causing peaks originating from distinct chemical environments to overlap.

**Figure 9:**
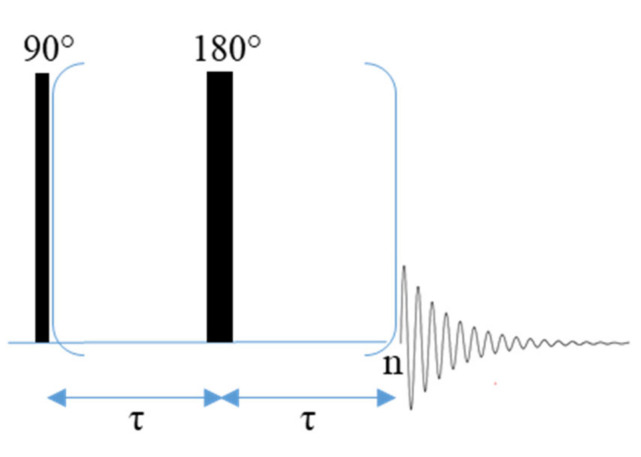
Carr-Purcell-Meiboom-Gill (CPMG) pulse sequence.

T_2_ relaxation can be measured using the spin-echo pulse sequence which is comprised of a 90° pulse followed by a delay time (*τ*), a 180° pulse, and a second delay period (*τ*) [86,87]. However, the Carr-Purcell-Meiboom-Gill (CPMG) pulse sequence (Table S1) is probably more commonly used to measure T_2_ relaxation [88,89]. This pulse sequence begins with a 90° pulse, a delay period (*τ*) and a 180° pulse as in the spin echo sequence, but also includes a train of 180° pulses at 2 *nτ* delay periods following the first 180° pulse which results in a series of spin echoes that occur at time 2 *nτ* (Figure 9) [89]. The echo train continues until the signal has decayed [88,89]. The advantage of using the CPMG pulse sequence over the spin echo pulse sequence is that the train of 180° pulses minimizes the potential loss of magnetization due to random spin diffusion, which prevents complete magnetization refocusing [89]. Both the spin-echo and the CPMG pulse sequences are used in some of the experiments that are discussed here.

In a single-pulse experiment, observed signal decay is caused by a combination of T_2_ relaxation and decay due to magnetic field inhomogeneity [29]. However, the spin echo pulse sequence allows losses due to field inhomogeneity to be recovered. Following the application of the 90° pulse, magnetic field inhomogeneity causes spins in regions of relatively high magnetic field to precess faster than spins that are in a region of relatively low magnetic field [29]. After the spins have evolved for time τ, the phases of magnetization of different regions are sufficiently different to cause a decrease in the overall magnetization. This is illustrated in equation (12) where T_2_^*^ is the observed decay, *T_2_* is the transverse magnetization, *γ* is the gyromagnetic ratio, and *ΔH_0_* is the inhomogeneity of the magnetic field.

(12) $$\frac{1}{T_{2}^{*}}=\frac{1}{T_{2}}+\gamma \Delta H_{0}\;\;\;\;$$

However, the spins in each individual region of the magnetic field are still coherent and precessing in the transverse plane [29]. This dephasing effect is then reversed by the application of a 180° pulse as all transverse spins are reflected in the direction of the applied pulse [29]. This reverses the motion of the spins and, because faster spins get de-phased at a faster rate, after the second delay period all spins are back in phase and the total magnetization reaches a maximum (yielding a spin echo) [88]. The height of the spin echo shows what the free induction decay would have been at a time of 2*π* if no field inhomogeneities were present [89]. For the CPMG pulse sequence, 180° pulses are repeated until the echo has completely decayed. In this case, T_2_ is calculated using equation (13) where *I(2 nτ)* is the signal after the n^th^ echo and *I(0)* is the initial signal.

(13) $$I\left(2n\tau \right)=I\left(0\right)e^{\left(\frac{-2n\tau }{T_{2}}\right)}\;\;\;\;\;$$

**Figure 10:**
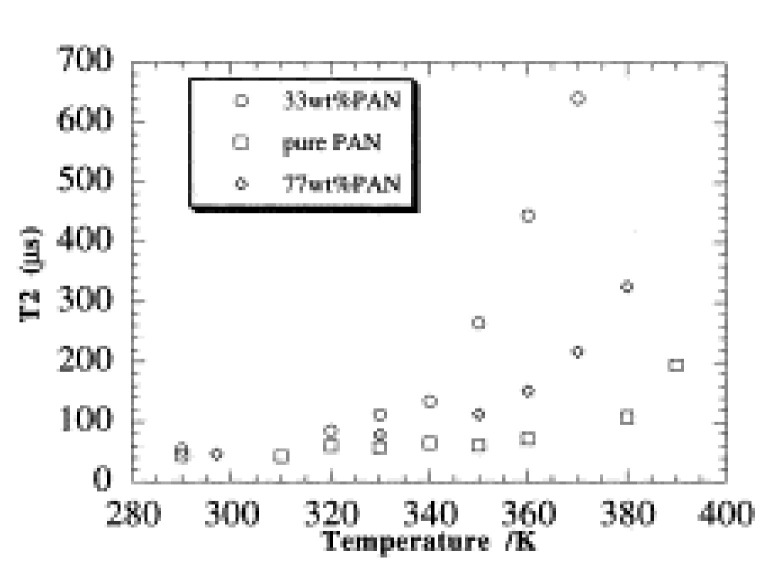
T_2_ relaxation as a function of temperature for pure polyacrylonitrile (PAN) and PAN electrolytes containing 30% and 67% by weight LiTf. T_g_ was designated as the temperature where T_2_ relaxation changed significantly which was indicative of significant polymer chain motion. Reprinted from [87]. Copyright 2000 with permission from Elsevier.

T_2_ relaxation, as determined using the spin-echo pulse sequence, has been used in a few instances in the analysis of polymer films as an alternative method for measuring the effects of electrolyte hydration and lithium salt addition [89,90]. This was the case in the work of Donoso et al. who compared *T*_2_ values in hydrated and anhydrous PEO-LiBF_4_ polymer electrolytes [90]. *T*_2_ relaxation was found to increase with hydration with the anhydrous film having a *T*_2_ of 45 μs and the hydrated film having a *T*_2_ of 130 μs [90]. The increase in T_2_ relaxation time was attributed to the fact that water is predicted to act as a plasticizer in this system by both increasing the mobility of the polymer chains and by increasing the volume of the mobile segments [90]. The fact that changes in polymer chain mobility can be observed via T_2_ relaxation was also used by Forsyth et al. as an alternative means of determining T_g_ in polyacrylonitrile (PAN)-LiTf films that were not amenable for analysis via DSC [89]. The T_g_ was identified as the temperature at which a large increase in T_2_, which was correlated with increased polymer chain mobility, was observed (Figure 10). In this case the pure polymer had a T_g_ of 107 °C, whereas the sample containing 67% LiTf had a T_g_ of 72 °C, showing that increased salt content increased polymer plasticity [89]. It is noted by the authors that the T_g_ value for the pure PAN sample is about 10 degrees higher than the standard DSC value. The above studies show that T_2_ relaxation can be used instead of or in combination with differential scanning calorimetry to elucidate the impact of temperature on polymer mobility and thermal properties.

The Carr-Purcell pulse sequence has been used to identify environments with different mobilities in polymer electrolytes. The ability to identify and characterize multiple species in the same sample is typically one of the advantages of using NMR spectroscopy over electrochemical techniques. This has been demonstrated in the context of T_2_ relaxation by several authors including Franco et al. who found that segments in a PEO polymer containing LiClO_4_ and 20 wt% carbon black have two distinct mobilities via ^1^H NMR [91]. The site with the longer T_2_ was attributed to the mobile polymer chains and the site with the shorter T_2_ was attributed to the immobile backbone [91]. A similar experiment was performed by Kwaks et al. who measured ^1^H T_2_ in polymer electrolytes comprised of poly(oligo oxyethylene methacrylate) and LiTFSI [92]. They found three separate T_2_ values which corresponded to the mobile side chain (T_2_ = 70 ms), the interface between the polymer chains (T_2_ = 10 ms) and the immobile polymer backbone (T_2_ = 1 ms) [92]. The characterization of three distinct lithium environments in a single system would not have been possible via most electrochemical methods. T_2_ relaxation was also used by Kidd et al. to identify two distinct lithium environments in PEO-based polymer electrolytes that are crosslinked to polyvinylidene fluoride (PVDF) filaments [93]. These materials were soaked in a mixture of ethylene carbonate, dimethyl carbonate, and lithium triflate and contained 10, 20, or 30 wt% pentaerythritol triacrylate (PETA). Two distinct lithium environments, one with a long T_2_ (195–310 ms depending on PETA content) and another with a shorter T_2_ (12–21 ms depending on PETA content) were identified by the authors [93]. The length of the lithium environment with short T_2_ was estimated at 3 μm based on a combination of the 2*τ* delay period and diffusion data from PFG experiments [93]. This finding, and the fact that the fraction of the long T_2_ environment increased from 26% to 79% when the PETA weight percent was increased from 10 to 30, allowed the environment with the short T_2_ being assigned to the PVDF filaments and the environment, with long T_2_ being assigned to the PEO between the filaments. The results of the above studies suggest that T_2_ relaxation measurements can be performed as a method of determining, among other things, the number of sites present in a sample, their mobilities, and their thermal properties.

#### 2.2.3. Linewidth

**Figure 11:**
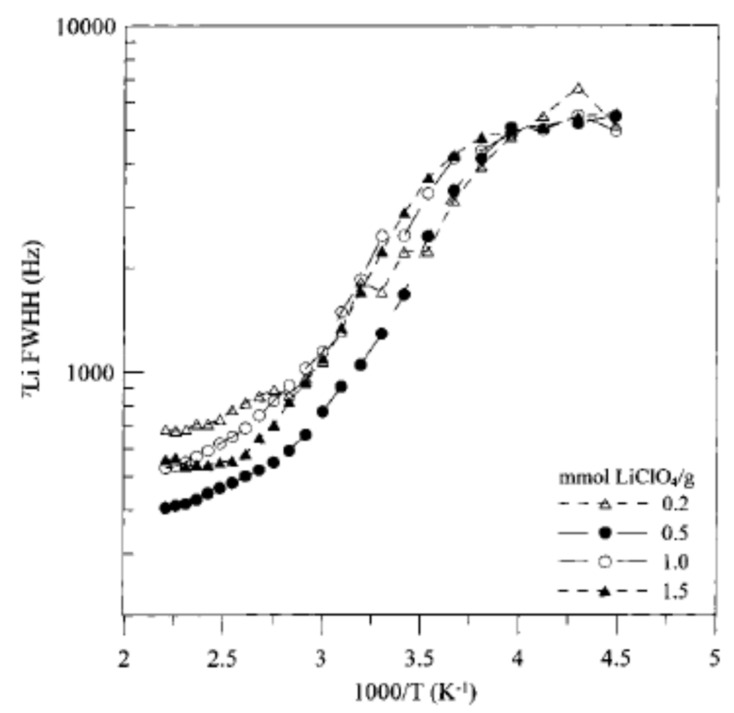
Arrhenius plot based on full width at half maximum (FWHM) measurement from a series of PEO-polydimethylsiloxane (PDMS) films doped with varying amounts of LiClO_4_. Reprinted with permission from [76]. Copyright 2002 American Chemical Society.

Similar to T_1_ or T_2_ relaxation, changes in spectral linewidth as a function of temperature can be compared to assess the relative mobility of species in a sample with narrower lineshapes indicating increased mobility [94,95]. The analysis involves fitting individual peaks in an NMR spectrum and measuring the full width at half maximum (FWHM) of each site. This technique is widely used to assess mobility as it does not necessarily require complex NMR experiments to be performed (Table S1). However, techniques such as magic angle spinning (MAS) and proton decoupling are often used to produce narrower lineshapes which can be more readily deconvoluted and fit. While changes in FWHM can be used to quantify the relative mobilities of sites in a sample under the same experimental conditions, this technique is not suitable for the direct quantitative comparison of ion dynamics when different NMR experiments are performed as experimental parameters can impact FWHM. However, linewidth analysis can be used to quantitatively assess site-specific ionic motion between samples and/or under different experimental conditions if changes in FWHM are used to calculate activation energy. Activation energies can be calculated from Arrhenius plots of FWHM as a function of temperature (Figure 11) and, like T_1_ relaxation, linewidth analysis can be coupled with electrochemical impedance spectroscopy measurements to describe the contributions of site-specific motions to global lithium transport [76,96,97].

Linewidth analysis is commonly used to assess the mobility of sodium sites in solid polymer electrolytes. However, as the ^23^Na quadrupolar moment can be significant (up to about 7 MHz), these interactions can significantly impact linewidth making it difficult to discern which contributions to the total linewidth come from temperature-dependent ion mobility and which are caused by second order quadrupolar effects [98]. In addition to impacting linewidth, quadrupolar interactions can also alter the chemical shift from its isotropic value [99]. Despite the potential drawbacks of the quadrupolar interaction in sodium-based systems, the quadrupolar contribution to the linewidth can reveal a significant quantity of information about the sodium sites’ immediate coordination environment.

**Figure 12:**
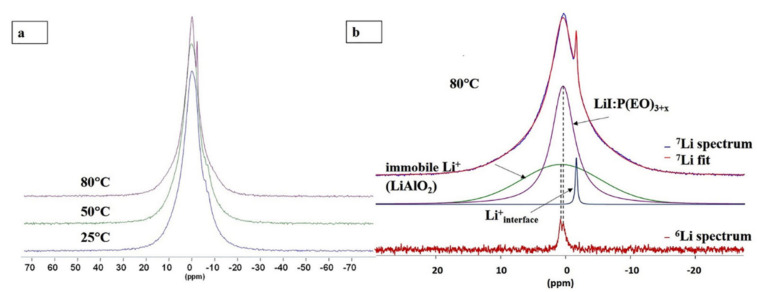
Variable temperature ^7^Li NMR spectra (7.1 T, 5 kHz) of a PEO-LiI polymer matrix containing LiAlO_2_ (**a**). Deconvolution of the spectrum at 80 °C (**b**). Reprinted from [101]. Copyright 2019 with permission from Elsevier.

Variable temperature lineshape analysis has been used to evaluate species mobility in complex polymer electrolyte systems [100]. An example of this is the use of ^7^Li NMR to evaluate differences in ion mobility in a polymer–gel electrolyte mixture that was comprised of polyacrylonitrile (PAN), polypropylene carbonate, ethylene carbonate and LiClO_4_ salt [94]. In this case, significant line narrowing was observed in the gel component while the peak corresponding to the PAN remained broad, signaling low ion mobility in the polymer component [94]. In addition to probing component mobility in polymer–gel systems, variable temperature linewidth analysis has also been used to evaluate PEO systems containing inert and active ceramic dopants [13,95,101]. Chung et al. prepared PEO-LiClO_4_ films containing 10 wt% TiO_2_ [95]. The addition of the ceramic nanoparticles resulted in an increase in the FWHM relative to the TiO_2_-free sample. This was attributed to decreased segmental motion of the polymer chains as a result of crosslinking between the polymer and the TiO_2_ nanoparticles [95]. Bonizzoni et al. used variable temperature ^7^Li NMR to analyze a PEO-LiTFSI system containing Li_1.3_Al_0.3_Ti_1.7_(PO_4_)_3_ (LATP) between 25 and 75 °C [13]. Coalescence between peaks corresponding to two lithiated sites in the ceramic indicated that the rate of lithium-ion exchange between these sites increased with temperature. [13]. The peak corresponding to LiTFSI narrowed indicating increased lithium mobility [13]. Menkin et al. used ^7^Li variable temperature NMR to evaluate the impact of LiAlO_2_ content on a PEO-lithium iodide (LiI) system [101]. It was found that increasing sample temperature resulted in peak narrowing between 25 and 50 °C for the peak corresponding to lithium ions in PEO and the appearance of a sharp peak (from a previously existing shoulder) at 80 °C which was assigned to the polymer–ceramic interface [101]. No changes in linewidth were observed in the peak corresponding to the bulk ceramic indicating that it did not participate in ion conduction (Figure 12) [101]. It can be seen that lineshape analysis can be particularly useful in distinguishing mobility at different sites in complex systems such as hybrid electrolytes where differences in ion dynamics between the polymer and ceramic components, as well as any interfaces, can be expected.

**Figure 13:**
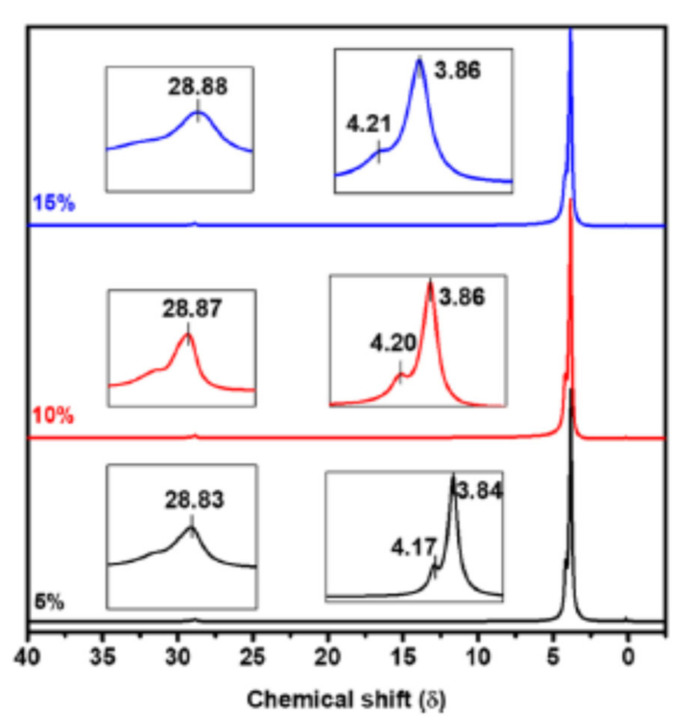
^1^H NMR spectra (9.4 T) of PEO-LiClO_4_ membrane containing various mass percentages of SiO_2_. Reprinted with permission from [102]. Copyright 2020 American Chemical Society.

In addition to its use in variable temperature NMR, lineshape analysis has also been used at constant temperatures to evaluate the effects of inert and active ceramic doping on the polymer matrix in hybrid electrolyte systems [102,103]. Xu et al. used ^1^H NMR to measure the hydrogen bonding interaction between a PEO-LiClO_4_ polymer matrix and an inert SiO_2_ nanoparticle dopant in order to evaluate the effects of SiO_2_ doping on PEO crystallinity [102]. They found that interactions between the SiO_2_ nanoparticles and the polymer chain reduced crystallinity in the PEO matrix. These interactions produced narrower lineshapes which indicated increased segment mobility which is typically associated with increased conductivity (Figure 13) [102].

Conversely, Zheng and Hu, who used ^6^Li NMR to analyze a PEO-LiTFSI electrolyte containing 20 and 50 wt% Li_7_La_3_Zr_2_O_12_ (LLZO) (an active ceramic) system, reported that increasing ceramic content increased the linewidth of the peak corresponding to the polymer as a result of decreasing crystallinity in the PEO polymer matrix [103]. As increased sample disorder may result in line broadening, the dual effects of sample disorder must be considered when evaluating NMR spectra. The types of interactions that are possible between the ceramic, either active or inert, means the polymer must therefore be well-understood to be able to determine whether changes in linewidth are indicative of changes in sample mobility or interactions between the polymer and the ceramic additive.

In addition to tracking lithium mobility, analysis of FWHM as a function of temperature was used by Lago et al. to compare the mobility of the TFSI anion in a PEO-LiTFSI electrolyte and an electrolyte system that was comprised of Al_2_O_3_ nanoparticles grafted to PEG and TFSI in a PEO-diglycidyl ether of poly(ethylene glycol) polymer matrix [104]. Analysis of ^19^F FWHM revealed that the TFSI anion was significantly more mobile in the PEO-LiTFSI electrolyte than in the grafted composite electrolyte [104]. This situation is of interest as lower anion mobility serves to increase cation transference number. Line width analysis is one of the most commonly used techniques to analyze differences in mobility of sodium environments in polymer electrolytes [83,98,99]. Wong and Zax tracked changes in FWHM for a hybrid electrolyte comprised of PEO, lithium salt and the sodium-containing silicate montmorillonite [98]. Measuring sodium FWHM between −73 and 77 °C revealed a general decrease in linewidth with temperature which suggests that the dipolar contribution to the linewidth dominates in this system. The observation of two low temperature plateaus in a plot of sodium linewidth as a function of temperature indicates that quadrupolar interactions also contribute to the observed linewidth [98]. Work by Forsyth et al. showed that the linewidth of a poly(ethylene oxide-co-propylene-oxide) polymer electrolyte containing NaCF_3_SO_3_ decreased from 4.2 to 0.6 ppm between −67 and 20 °C [99]. This is indicative of increased sodium mobility in this system and suggests that dipolar contributions to the linewidth also dominate in this system. In addition to affecting FWHM, the quadrupolar interaction can also cause the observed chemical shift to differ from its isotropic value. This phenomenon was evaluated by Spindler and Shriver in a poly(methyl-hydrosiloxane)-monomethyl ether poly(ethylene glycol) co-polymer that contained Na[SO_3_C_3_F_3_] [105]. The ^23^Na chemical shift was observed to become more positive when either sample temperature or salt content were increased [105]. This observation was attributed to increased interaction between cations and anions in this system.

### 2.3. Exchange Spectroscopy

Like relaxation and linewidth analysis, exchange spectroscopy (EXSY) can be used to quantify changes in ion motion as a function of sample temperature or doping. EXSY is typically used to identify site-specific exchange processes and provides quantitative data including rates of chemical exchange and activation energies for these processes. In one dimensional NMR spectra, chemical exchange is typically manifested as coalescence where the approximate rate of exchange can be evaluated based on the degree of peak overlap: minimal overlap indicates slow exchange, significant overlap with two sites still resolvable indicates intermediate exchange and complete overlap with line narrowing (motional exchange counteracts peak broadening) indicates fast exchange [106].

**Figure 14:**
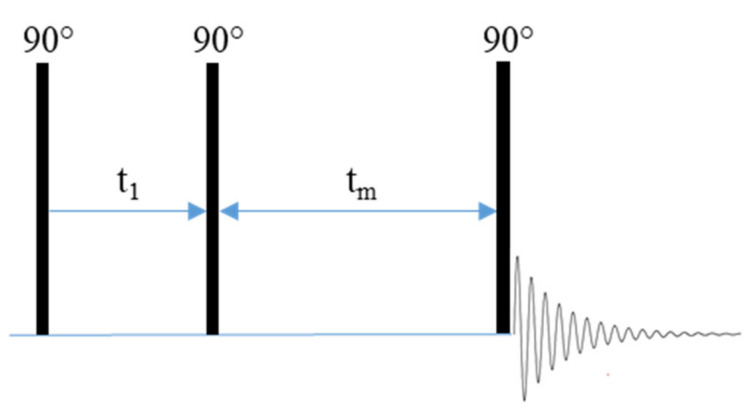
Exchange spectroscopy (EXSY) pulse sequence.

**Figure 15:**
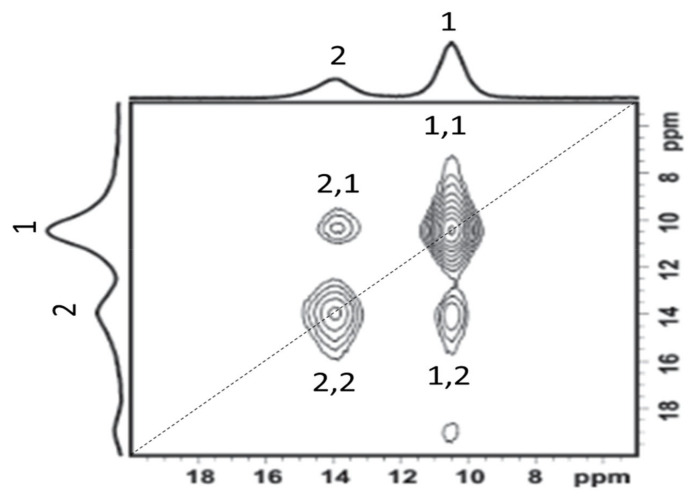
Sample EXSY spectrum. Diagonal peaks (1,1 and 2,2 along the dotted line) represent the original position of each spin and have the same frequency coordinates on both axes. Cross peaks (1,2 and 2,1) represent exchanged spins and have different frequency coordinates on each axis.

EXSY is a two-dimensional NMR experiment that is generally used to analyze systems undergoing slow and intermediate exchange processes [106]. Slow and intermediate exchange rates correspond to processes that occur prior to coalescence where the rate of exchange is less than the difference in Larmor frequency between the two sites [106]. The EXSY pulse sequence (Figure 14) is comprised of three 90° pulses (Table S1). The first pulse frequency labels all spins in the system, which are then allowed to evolve over a variable delay period *t_1_* [107]. The second pulse inverts all spins which relax and possibly undergo chemical exchange during the mixing time *t_m_* and are observed following the application of a third pulse [107]. All spins maintain the labeling that was created by the first pulse which causes signals related to chemical exchange between sites to appear as off-diagonal cross peaks. Off-diagonal peaks have different coordinates on each frequency axis in the two-dimensional spectrum, whereas diagonal peaks (same coordinates on each axis) represent the fraction of spin which did not undergo chemical exchange (Figure 15) [107]. Multiple exchange processes are represented by multiple sets of cross peaks. Quantitative data from EXSY experiments is obtained by fitting the integrated area of the cross peaks and the diagonal peaks. The cross peak area is normalized with respect to the diagonal peak area and can be plot as a function of mixing time to extract exchange rates [107]. Arrhenius plots of exchange rates can be fit to obtain activation energies for individual exchange processes. These activation energies are site specific and can be compared to electrochemical data to determine which local exchange processes do, and do not, contribute to long range conductivity. However, a disadvantage of obtaining kinetic parameters via EXSY is that the experiment is time consuming since two dimensional experiments must be performed at several mixing times for each temperature to obtain reliable rates of chemical exchange.

A first disadvantage to using EXSY to measure chemical exchange is that the exchange rate must be equal to or greater to the spin-lattice relaxation time [108]. This limitation can result in significant constraints when ^23^Na NMR is considered as the quadrupolar interaction in many sodium environments causes T_1_ relaxation to occur more quickly relative to T_1_ relaxation in ^7^Li systems [47]. Chemical exchange processes that occur on a timescale that is slower than the spin-lattice relaxation cannot be observed via ESXY as cross peaks are not produced during the mixing time, t_m_ in Figure 15 [108]. A second disadvantage that is relevant to the use of EXSY to analyze the chemical exchange of quadrupolar nuclei is that quadrupolar interactions typically cause line broadening as a result of second order effects not being attenuated by regular MAS spectroscopy [109]. Significant line broadening has previously been shown to obscure otherwise observable cross peaks by decreasing peak separation and therefore increasing the minimum correlation time for a motional process to produce observable cross peaks [110].

In the analysis of solid polymer electrolytes, EXSY has been used to demonstrate polymer mobility and to establish lithium conduction pathways in solid polymer and hybrid electrolytes. To this end, Liu et al. used ^13^C EXSY to analyze polymer chain dynamics in a PEO-LiAsF_6_ system where the impact of various molecular weights of PEO on segmental motion was evaluated [111]. Analysis of the two-dimensional spectrum revealed cross peaks that were consistent with polymer chain diffusion [111]. The cross peaks that were observed between pairs of carbon atoms were consistent with forward and backward chain motion with the integrated area of the cross peaks being representative of the rate of this motion [111]. Chain diffusion was found to increase with PEO molecular weight as it was associated with amorphous regions in the polymer matrix which remain small at low molecular weights [111].

**Figure 16:**
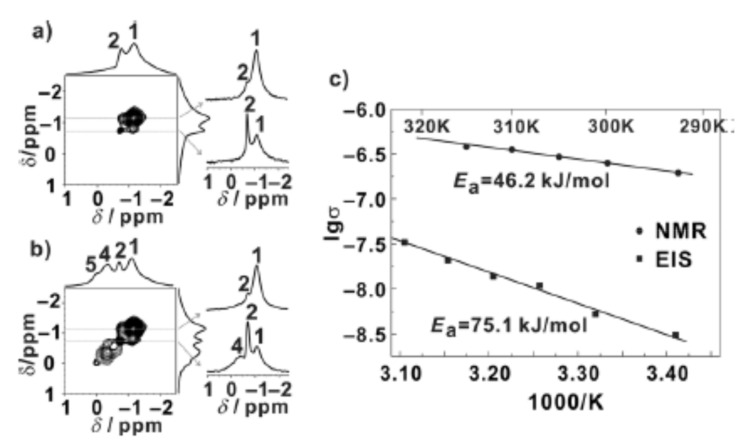
^7^Li EXSY spectra of PEO-LiAsF_6_ in cyclodextrin nanochannels (14 T, 5 kHz MAS) with a mixing time of 300 ms. Spectrum (**a**) was collected with no dipolar coupling and showed correlation between the PEO and the interface. Spectrum (**b**) was collected with dipolar coupling where correlation between the interface and the cyclodextrin layer is observed. Activation energies calculated based on EXSY NMR data and electrochemical impedance spectroscopy (EIS) data (**c**). Reprinted with permission from [112]. Copyright 2014 Wiley and Sons.

In addition to its use in the characterization of polymers, ^7^Li EXSY was also used to determine which lithiated environments are involved in ion conduction in a PEO-LiAsF_6_ system that is encased in a series of α-cyclodextrin nanochannels [112]. Prior to performing the two-dimensional experiment, five lithium environments were identified via one dimensional NMR techniques: Li-1 was attributed to lithium ions in PEO, Li-2 was attributed to interfacial lithium between PEO and the cyclodextrin nanochannels, Li-3 was attributed to “free” lithium ions, and Li-4 and Li-5 were attributed to lithium ions in the cyclodextrin nanochannels [112]. Cross peaks were found between Li-1 and Li-2 as well as between Li-2 and Li-4, suggesting that lithium ions pass through an interfacial layer when they are transferred between PEO and the nanochannel (Figure 14) [112]. The lithium exchange rate that was calculated from the EXSY data was greater than the measured conductivity which suggests that not all local-scale motional processes contribute to long range lithium conductivity in this system [112]. However, the activation energy that was calculated via EXSY was lower than that obtained via EIS indicating additional energy barriers for long-range lithium motion in this system (Figure 16) [112]. This work demonstrates that EXSY can be used to characterize several exchange processes in a single system provided that each signal in the NMR spectrum has been previously well-characterized. It also illustrates the role that many species can play in local-scale ion motion, which is not generally characterizable by electrochemical methods.

**Figure 17:**
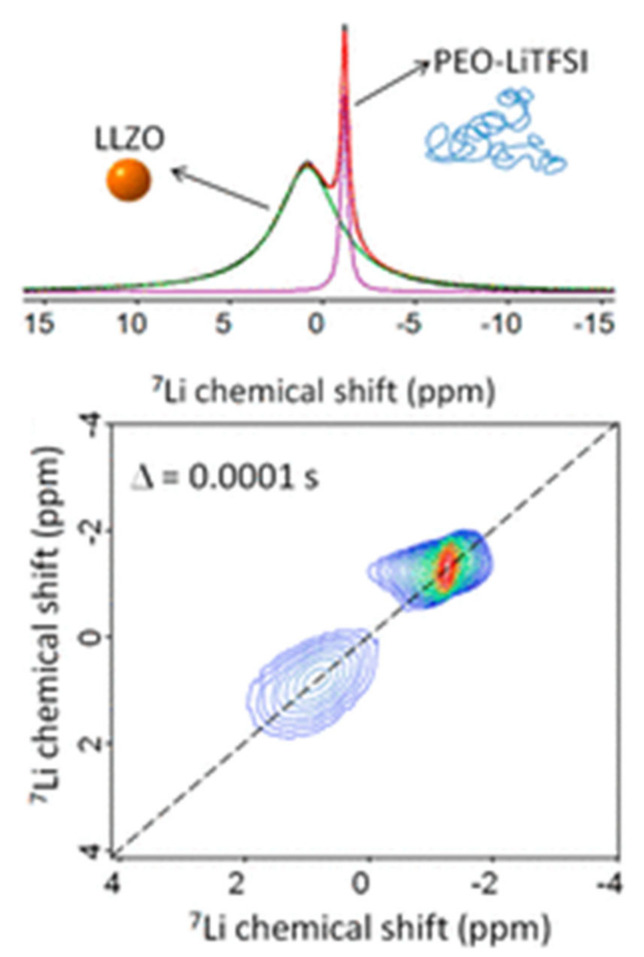
^7^Li one dimensional and EXSY spectra (14 MHz, 20 kHz MAS) of PEO-LiTFSI containing LLZO (Li_7_La_3_Zr_2_O_12_). Reprinted with permission from [114]. Copyright 2019 American Chemical Society.

EXSY is not limited to homogeneous systems and, as such, was also used to measure lithium exchange between the polymer matrix and active ceramic additives in hybrid systems. Zheng et al. analyzed a PEO-LiClO_4_ membrane containing LLZO, an active ceramic [113]. Their 1D ^7^Li experiments identified peaks attributed to the LiClO_4_ in the polymer matrix, LLZO and an interfacial layer [113] The EXSY experiments revealed cross peaks between the polymer and the interfacial layer [114]. No cross peaks were observed between LLZO and the polymer or LLZO and the interface [113]. These results suggest that lithium-ion conduction does not occur directly through the ceramic in this system. Conversely, the use of EXSY by Zagórski et al. to study a PEO-LiTFSI system containing LLZO revealed that no interface layer is formed, and that lithium-ion exchange instead occurs between the ceramic and the polymer directly (Figure 17) [114]. However, electrochemical impedance spectroscopy studies that were performed on this system suggest that it is not lithium exchange between the polymer and the ceramic, but the motion of lithium ions through the polymer, that is responsible for the majority of the lithium conductivity in this system [114]. The differences in the role of the polymer–ceramic interface in lithium conduction pathways in these materials could be a result of differences in LLZO content. The study by Zheng et al., where an interface was observed, involved samples containing 50 wt% LLZO while the samples used in the work of Zagórski et al. contained 10 wt% LLZO [113,114]. It is possible that the lower LLZO content of the samples in the latter study resulted in an insufficient quantity of interfacial lithium to detect. Additionally, lithium conduction is predicted to proceed primarily through the polymer matrix in hybrid systems with low ceramic loading [103].

### 2.4. Dipolar Coupling

#### 2.4.1. Cross Polarization

Like EXSY, cross polarization NMR spectroscopy (Table S1) can be used to measure correlation between chemical environments in complex systems. However, instead of relating environments based on chemical exchange, cross polarization is used to identify sites based on the strength of the heteronuclear dipolar coupling interaction that exists between nuclei. The cross-polarization experiment involves the transfer of magnetization from an abundant spin to a dilute spin [115]. The magnetization transfer happens as a result of the heteronuclear dipolar coupling interaction which is a distance-dependent through-space interaction that arises as a result of the interacting magnetic dipole moments of proximal nuclei [116]. As a result, stronger signals are indicative of stronger dipolar couplings, whereas weaker signals indicate weaker dipolar couplings. The main advantages of the technique are improved sensitivity for low abundancy nuclei (up to the ratio of gyromagnetic ratios) and a reduction in experimental time as the T_1_ of the abundant spin, which is generally shorter, is used during the experiment [115].

**Figure 18:**
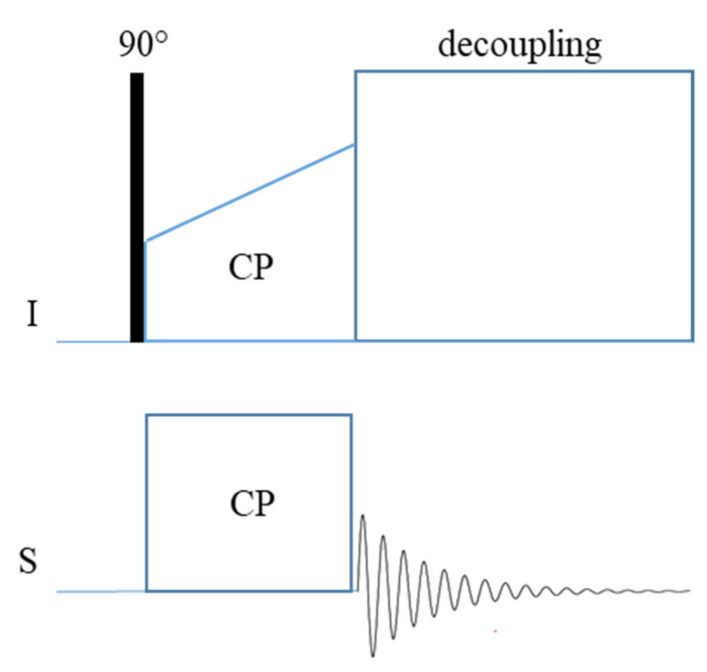
Cross polarization pulse sequence for the transfer of magnetization between an abundant spin (I) and a dilute spin (S).

The cross-polarization experiment (Figure 18) begins with the application of a 90° pulse on the abundant spin which is followed by a pulse that spin locks the magnetization of the abundant spin along the y-axis [115]. During this time, a pulse of same length is applied on the dilute spin which puts both magnetization vectors along the y-axis such that spin exchange can occur [115]. After the spin lock pulse, the radio frequency on the dilute spin is turned off to allow for signal acquisition while the abundant spin is decoupled [115].

For spin exchange to occur, the Hartmann-Hahn matching condition (Equation (14)), where both nuclei have equal rates of precession and equal effective energies, must be met [117]. This condition is obtained by setting the effective field (*B_1_*) on each channel such that the difference between the product of the effective magnetic field and the gyromagnetic ratio (*γ*) is equal to n times the MAS rate in kilohertz where *n* is equal to ±0, 1, 2…

(14) $$\gamma_{I}B_{1I}-\gamma_{S}B_{1S}=nMAS\;\;\;\;\;\;$$

As magnetization transfer between spins is dependent on the strength of the dipolar coupling interaction, the resultant spectral intensities can be used to gauge distances between chemical environments (stronger signals are indicative of spin environments being closer together) and site mobility (increased motion decreases the strength of the dipolar coupling interaction).

Although CP NMR has yet to be used to measure sodium-ion or sodium-polymer interactions in polymer or hybrid electrolytes, it must be noted that CP NMR spectroscopy is possible between quadrupolar and non-quadrupolar nuclei as well as between two quadrupolar nuclei. In the case of cross polarization between a spin 3/2 or 5/2 nucleus and a spin 1/2 nucleus, the Hartmann-Hahn condition (Equation (14)) may be satisfied by matching the RF field strength of the spin 1/2 nucleus to the nutation frequency of the quadrupolar nucleus [118]. This condition is generally achieved by preceding the CP portion of the pulse sequence with a technique used to generate multiple quantum coherence from the quadrupolar nucleus such as multiple quantum magic angle spinning [118]. In the case of two quadrupolar nuclei, RF irradiation during MAS induces spin state mixing which makes it extremely difficult to achieve the simultaneous spin-locking that is required to satisfy the Hartmann-Hahn condition (Equation (14)) [119]. However, Puls and Eckert were able to perform ^23^Na-^7^Li CPMAS on the mixed cation glass [(Li_2_O)_x_(Na_2_O)_1-x_]_0.3_[B_2_O_3_]_0.7_ by first acquiring a detailed understanding of the quadrupolar coupling behaviour of both nuclei by performing multiple quantum excitation and satellite transition MAS spectroscopy prior to commencing any CPMAS experiments [119].

Cross polarization NMR experiments have been performed to monitor nanochannel formation in a PEO-LiAsF_6_ α-cyclodextrin electrolyte system [112,120]. ^1^H-^13^C cross polarization experiments showed that the conformation of the glucose units in the cyclodextrin changed upon nanochannel formation and was used as an indicator to demonstrate that this polymer configuration was achieved [112,120]. Additionally, in the study by Yang et al., a combination of isotope labelling, and cross polarization NMR was used to assign lithium environments to the PEO moiety or the nanochannel [112]. This was done by labeling the PEO moiety with deuterium such that the lithium environments that were visible via ^2^H-^7^Li cross polarization NMR could be assigned to the PEO whereas environments that were visible via ^1^H-^7^Li cross polarization NMR could be assigned to the nanochannel. Lithium environments that were not observed in either experiment were deemed to be free lithium as larger lithium-hydrogen distances would reduce the visibility of these sites as a result of reduced dipolar coupling interactions [112]. Limited proton exchange between the nanochannel and the PEO domains was essential for allowing deuterium enrichment to be used as a method of distinguishing between lithium environments in this system [112]. Isotope labeling experiments will be discussed in more detail in the following section.

**Figure 19:**
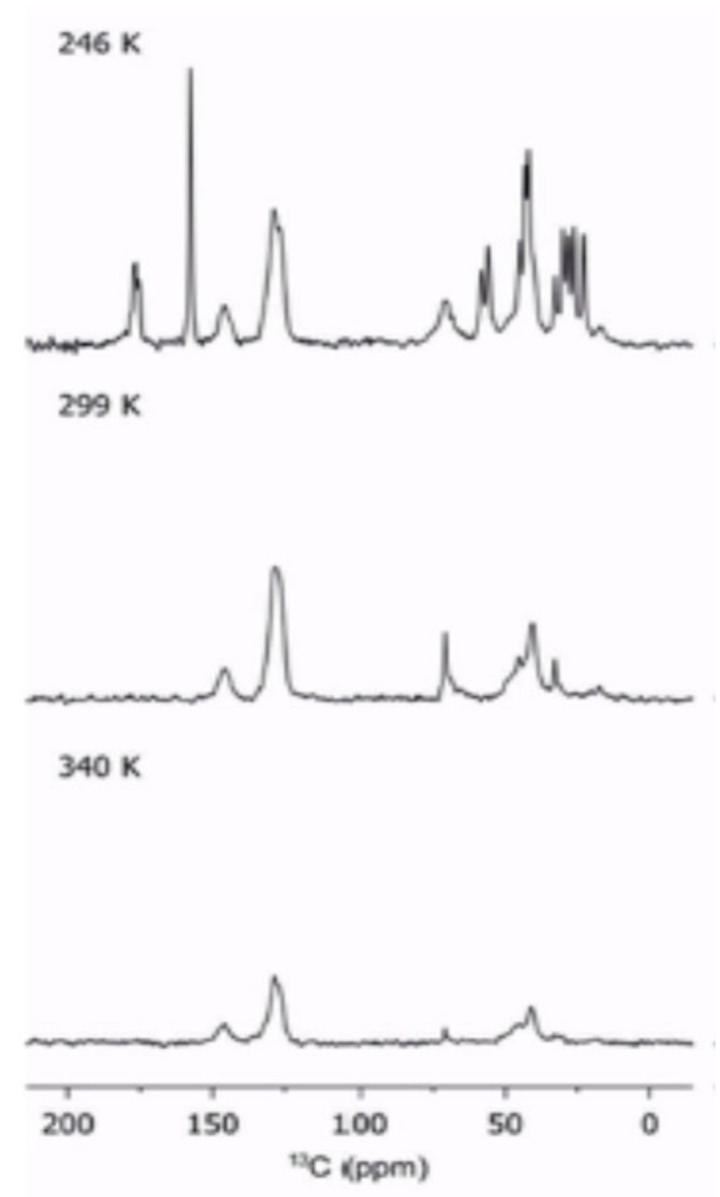
^1^H-^13^C cross polarization NMR spectra of a polystyrene PEG-methylmethacrylate co-polymer at various temperatures (9.4 T, 20 kHz MAS). Magnetochemistry 2018 [31].

The intensity of cross polarization spectra can also be used to gauge ion mobility as increased mobility tends to reduce the strength of the dipolar coupling interaction [121]. ^1^H-^13^C cross polarization NMR has been used to compare the segmental mobilities of different polymer segments in PEG-based co-polymers. For example, in their analysis of a polystyrene PEG-methylmethacrylate co-polymer system, Daigle et al. found that the polystyrene backbone is less mobile than the PEG-methyl methacrylate sidechains as a result of the presence of attenuated peaks in a ^1^H-^13^C cross polarization spectrum [31]. However, as was determined via cross polarization NMR by Lin et al. in their analysis of a PEG-PDMS co-polymer, the mobility of the sidechain groups varied based on the degree of lithium salt loading [76]. Polymer side chains containing more lithium salt tended to be less mobile as a result of stronger ethylene oxide lithium salt interactions. Additionally, increased sample temperature, which contributes to increased polymer mobility resulted in attenuation of the ^1^H-^13^C cross polarization signal (Figure 19) [31]. These works show that cross polarization NMR provides enhanced characterization of a complex polymer system relative to what is possible with most single nucleus experiments. Additionally, cross polarization is a one-dimensional experiment, meaning that a detailed characterization of the polymer structure and site interactions can be achieved more quickly than is possible using most two-dimensional techniques.

#### 2.4.2. Rotational Echo Double Resonance (REDOR)

**Figure 20:**
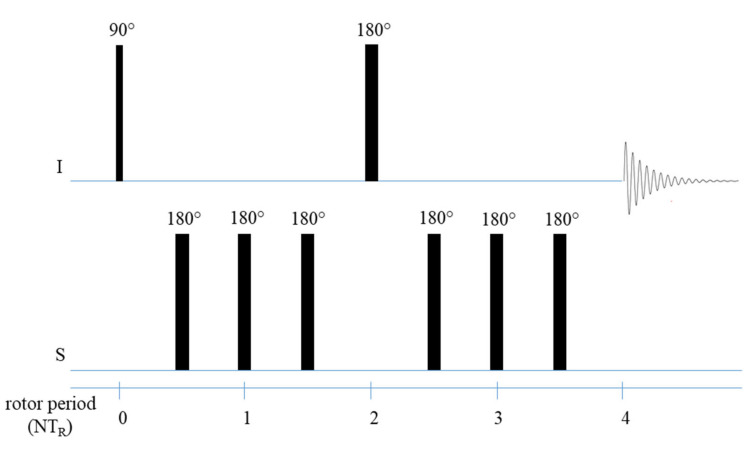
Rotational echo double resonance (REDOR) pulse sequence.

Rotational echo double resonance (REDOR) experiments (Table S1) are similar to cross polarization experiments in the sense that dipolar coupling interactions are used to transfer magnetization between two spins [122]. However, the REDOR experiment is generally used to provide more specific structural information as it yields site-selective dipolar coupling information for isolated spin pairs which allows internuclear distances to be determined [122,123]. The dipolar coupling interaction, which is normally averaged out during MAS conditions, is re-introduced during the pulse sequence (Figure 20) by the application on the non-observed spin S of a train of 180° pulses that serves to invert the sign of the dipolar Hamiltonian making the average interaction non-zero [123]. The detected spin I is observed by the application of a rotor synchronized spin echo pulse sequence (Figure 20) where the 90° pulse is applied at the beginning of the experiment and the 180° pulse is applied following 2N rotor periods (T_r_) where N is the number of cycles [122,124].

Dipolar coupling constants for isolated spin pairs can be extracted from REDOR spectra by plotting the ratio of the REDOR intensity (*ΔS*) and the normal rotational angle intensity (*S_0_*) as a function of the dipolar evolution time as the REDOR build up curve has a universal shape in this scenario [122,123,124]. The intensity of the REDOR spectrum (*ΔS*) is expected to decrease with increasing strength of the dipolar coupling interaction. In multi-spin systems, dipolar build up curves tend to be more complex as they are influenced by the shape and geometry of the system [123,125,126]. However, the strength of the dipolar coupling interaction can still be approximated by fitting the early part of the REDOR build up curve before these factors have a significant influence on the shape of the curve [125,126]. In addition, the fact that the examples that are discussed here involve dipolar coupling between protons and quadrupolar ^7^Li, the impact of the quadrupolar coupling interaction can also be important. However, it has been determined that the use of the pulse sequence shown in Figure 20, coupled with the low strength of the quadrupole moment in ^7^Li results in the quadrupolar coupling being cancelled out as it has an equal effect on both *ΔS* and *S_0_* which allows Equation (15), where *I* is the spin of the I spin, *m* is the magnetic quantum number, *NT_r_* is the dipolar evolution time, and *M_2_* is the second moment which can be extracted from the *ΔS*/*S_0_* using a parabola fit [123].

(15) $$\frac{\Delta S}{S_{0}}=\frac{1}{2I+1}\left(\overset{I}{\underset{m=-1}{\sum }}{\left(2m\right)}^{2}\right)\frac{1}{\pi^{2}\left(I+1\right)I}{\left(NT_{R}\right)}^{2}M_{2}\;\;\;\;\;\;\;$$

Voigt and Wüllen used REDOR to assign lithium environments in polymer electrolyte samples that were comprised of PAN and LiBF_4_ [127]. Two samples with differing proportions of LiBF_4_ (67 wt% and 14 wt%) were analyzed. ^1^H-^7^Li cross polarization spectra revealed two lithium sites, at −1.3 ppm and −2.0 ppm that were coupled to protons. As the analyzed samples were prepared by solution casting in deuterated DMSO, these sites were attributed to interactions between the lithium cations and the polymer [127]. The signal at −1.3 ppm showed no attenuation between the spin echo spectrum and the REDOR spectrum indicating limited dipolar coupling interactions. This was attributed to fast lithium motion decreasing the strength of the dipolar coupling interaction [127]. This site was therefore assigned to mobile lithium cations. The significant attenuation of the signal at −2.0 ppm was observed when the spin echo spectrum was compared to the REDOR spectrum indicating stronger dipolar coupling interactions [127]. A plot of *ΔS*/*S*_0_ as a function of recoupling time revealed that the dipolar coupling in the sample containing 67 wt% LiBF_4_ was 1.4 kHz and that the dipolar coupling in the sample containing 14 wt% LiBF_4_ was 1.8 kHz indicating that having a greater percentage of PAN increases interactions between the salt and the polymer [127]. Voigt and Wüllen also performed a ^13^C-^7^Li REDOR experiment using a PEO-LiBF_4_ polymer electrolyte (36 wt % PEO and 4 wt% LiBF_4_) which also contained 60 wt% succinonitrile [128]. The spin echo experiment revealed two ^13^C signal that were correlated to lithium. One signal at 66.2 ppm exhibited a significant REDOR response and was attributed to polymer matrix containing the lithium salt [128]. The other signal at 70.7 ppm did not experience attenuation during the REDOR experiment and was attributed to an amorphous succinonitrile-based phase with little to no dipolar coupling interactions as a result of fast ion motion in the plasticized polymer [128]. As REDOR signal intensity is dependent on the strength of dipolar coupling interactions, it can be used, as shown here, to determine how changes in electrolyte composition affect polymer–salt interactions and predict subsequent ion mobilities. It is also anticipated the variable temperature REDOR experiments could be performed to determine how changes in temperature impact polymer–salt interactions, and therefore ion mobility.

### 2.5. Isotope Enrichment

As mentioned in the ^1^H-^13^C cross polarization analysis of PEO in cyclodextrin nanochannels performed by Yang et al., isotope enrichment can be performed to enhance other types of NMR experiments [112]. Isotope enrichment experiments are typically performed to highlight specific regions of a sample, as was demonstrated above via the use of ^2^H doping to distinguish hydrogen in PEO and cyclodextrin environments [112]. Since NMR spectroscopy can be performed using any isotope where the nuclear spin is greater than zero, there exists isotope enrichment options for several nuclei. The most common isotope enrichment experiment that is performed in solid polymer electrolyte research is replacing ^7^Li with ^6^Li. Often, these experiments are not performed using enriched samples as ^6^Li has a natural abundance of 7.6% [43]. A particular advantage of performing ^6^Li NMR as opposed to ^7^Li NMR is that ^6^Li nuclei experience less homonuclear dipolar coupling than ^7^Li nuclei as a result of its lower gyromagnetic ratio with respect to that of ^7^Li (3.937 vs. 10.398 ×10^−7^ rad s^−1^ T^−1^) [43]. This results in narrower line shapes, making it easier to identify individual chemical environments in a sample. This technique was used by Zheng et al. to observe a peak attributed to a lithiated polymer–ceramic interface in a PEO-LiClO_4_ sample containing LLZO particles, an active ceramic [113].

**Figure 21:**
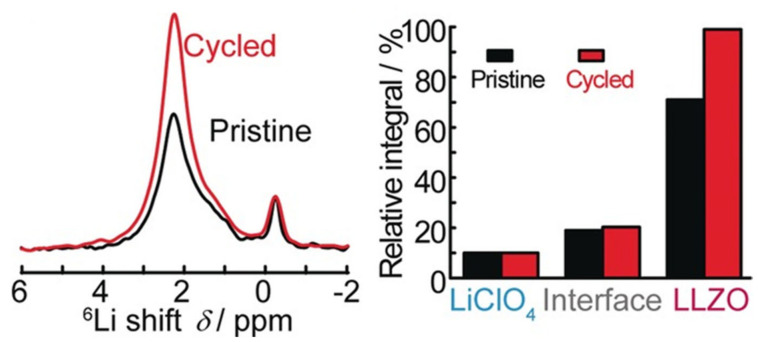
^6^Li foil cycling experiment performed on a PEO-LiTFSI system containing LLZO (14.1 T, 25 kHz MAS). Reprinted with permission from [113]. Copyright 2014 Wiley and Sons.

^6^Li NMR is used in the analysis of solid-state polymer electrolytes to track the motional pathways of lithium in the electrolyte layer. This is done by cycling against ^6^Li foils such that the regions that experience ^6^Li enrichment post-cycling are identified as being a part of the lithium conduction pathway. This is because, during cycling, ^6^Li ions are stripped from the foil and travel through the electrolyte. These replace existing ^7^Li ions in the polymer matrix and/or ceramic and leave a trail of ^6^Li-enhanced regions showing the lithium migration pathway through the electrolyte (Figure 21) [113]. The main strength of this method is that the regions involved in lithium conduction can be observed directly. However, this technique is most effective in systems where motion between domains (i.e., lithium in the polymer and lithium in the ceramic) is limited as some lithium exchange can occur under ambient conditions and would result in a slight ^6^Li enrichment of the whole system.

This experiment has been performed by several researchers using different polymer ceramic blends [113,129,130,131,132,133]. The exact conduction pathway seems to be dependent on many factors including: type of polymer used, type of ceramic used, and amount of ceramic in the system. This is likely a result of the fact that it is possible for lithium to travel through the polymer only, through the interface that is produced when ceramic is introduced into a polymer matrix or through the ceramic additive only [103].

In this vein, it has been suggested that in systems which contain high quantities of ceramic, lithium conduction occurs primarily through the ceramic particles. This was observed by Zheng et al. in a PEO-LiClO_4_ system containing 50 wt% LLZO, an active ceramic [113]. Yang et al. prepared polymer electrolytes from polyvinylidene fluoride-hexafluoropropylene and LiTFSI that contained 30 wt% Li_0.33_La_0.557_Ti_1-x_Al_x_O_3_ nanowires [133]. Following cycling with ^6^Li foil, the majority of the lithium signal (87.9 mol% by peak area) was located in the ceramic [133]. At lower ceramic contents, lithium conduction was observed to be more likely to occur through the interface that forms as a result of interactions between ceramic additives and the polymer matrix. In a PEO-LiTFSI system containing 25 wt% of the active ceramic LiZr_2_(PO_4_)_3_ (LZP), it was found that the most significant ^6^Li enrichment occurred in the disordered polymer environment that was believed to surround the ceramic particles [131]. Cycling a PEO-succinonitrile-LiTFSI system containing the active ceramic Li_1+x_Al_x_Ge_2−x_(PO_4_)_3_ (LAGP) against ^6^Li foils revealed the appearance of a new lithium environment whose integrated intensity increased with increasing LAGP doping suggesting that lithium conductivity occurs through this interfacial environment [130]. It is believed that lithium conduction occurs primarily through the polymer–ceramic interface at lower levels of ceramic doping as there is not sufficient ceramic volume to form a continuous ceramic network through the sample. At even lower ceramic content, lithium conduction was found to occur primarily through the polymer matrix. This was demonstrated via a ^6^Li foil cycling experiment by Xu et al. who used a PEO-LiTFSI system containing 10 wt% of the active ceramic Li_3/8_Sr_7/16_Zr_1/4_O_3_ (LSTZ) and Yang et al. who used a PAN-LiClO_4_ system containing 5% LLZO nanowire [131,132]. These experiments suggest that cycling a system against an ^6^Li foil is an effective way of identifying ion conduction pathways in hybrid electrolytes with various levels of ceramic loading. It is anticipated that similar results could be obtained using ^6^Li-enriched ceramics as changes in spectral intensity, which would indicate whether/to what extent these species participate in ion transport.

## 3. Future Work

Despite the advantages of NMR spectroscopy for the analysis polymer and hybrid electrolytes, improvements can still be made to better characterize these materials. To this end, an additional technique, fast field cycling NMR relaxometry, in which the variance in T_1_ relaxation changes with changes in the magnetic field is used to calculate correlation times and surface diffusion associated with mobile species will be discussed here. This has had limited use in the analysis of polymer or hybrid electrolytes. However, this technique has been previously used to analyze segmental motion in polymeric materials and ion mobility in confined ionogels [134,135,136] and therefore has the potential to be employed in future studies in this field. These techniques can be tailored to selectively analyze specific chemical environments in a sample via sample synthesis or experimental setup allowing for even further selectivity during analysis.

Future work in the analysis of polymer and hybrid electrolytes for use in all solid-state batteries could also focus on sodium-based batteries as the high availability and high energy density of sodium is creating increased interest in these devices. However, the higher quadrupole moment, relative to Li, can complicate the analysis of these systems by NMR spectroscopy. Future studies to investigate the ion mobility in and the molecular structure of sodium electrolytes could include NMR techniques such as multiple quantum magic angle spinning and satellite transition magic angle spinning, which are specifically used to determine the number of sites present, along with their quadrupole parameters. These experiments would provide information on the nuclear environments of sodium species and inform line shape fitting. Quadrupolar line shape fitting could then be used to determine the impacts of electrolyte structure and external factors, such as sample temperature on nuclear environments and molecular dynamics in these electrolytes.

## 4. Conclusions

The above examples show that solid-state NMR spectroscopy is a useful method for the analysis of molecular structure and ion dynamics in solid polymer and hybrid electrolytes. PFG NMR, which is commonly used to measure transference number in electrolyte systems, was shown to be good at characterizing local-scale ion mobility but typically worse than EIS-based measurements at describing long-range diffusion as it cannot distinguish between free ions and aggregates. Although not widely used in the analysis of solid polymer electrolytes, electrophoretic NMR is an experimentally demanding method to obtain species-specific transference numbers with accuracy that is not limited to dilute solutions. The ability of NMR based techniques to accurately describe local-scale motions that are not necessarily correlated with long-range processes can be observed in the use of variable temperature-assisted techniques for the quantification of mobility and the calculation of site-specific activation energies: T_1_ relaxation, linewidth analysis, and EXSY. Solid-state NMR can also be used to determine molecular structure and mobility simultaneously. This has been done via cross polarization and isotope replacement experiments. All things considered, solid-state NMR, alone or coupled with electrochemical analysis, is a powerful technique for characterizing molecular structure and ion mobility in polymer and hybrid electrolyte materials.

## Data Availability

This study did not generate any data.

## Supplementary Materials

The following are available online at https://www.mdpi.com/article/10.3390/polym13081207/s1, Table S1: Requirements for the analysis of solid polymer electrolytes using the NMR spectroscopy experiments that are presented in the text.
