# Supplementary material for: Use of Solid-State NMR Spectroscopy for the Characterization of Molecular Structure and Dynamics in Solid Polymer and Hybrid Electrolytes
Source: Polymers (Basel). 2021 Apr 8;13(8):1207. doi: 10.3390/polym13081207 (PMC8068304; doi:10.3390/polym13081207)
Supplement: Supplementary file 1 [file polymers-13-01207-s001.pdf]

**Supplementary Information**

Gabrielle Foran[1*], Nina Verdier[1], David Lepage[1], Cédric Malveau[1], Nicolas Dupré[2] Mickael Dollé[1*]

Use of Solid-State NMR Spectroscopy for the Characterization of Molecular Structure and Dynamics in Solid Polymer and Hybrid Electrolytes

1. Université de Montréal, Department of Chemistry, 1375 Avenue Thérèse-Lavoie-Roux, Montréal, Québec, Canada, H2V 0B3

2. Université de Nantes, CNRS, Institut des Matériaux Jean Rouxel, IMN, F-44000 Nantes, France

* Corresponding Authors

Gabrielle Foran: gabrielle.foran@umontreal.ca

Mickael Dollé: mickael.dolle@umontreal.ca

One of the objectives of this article is to provide a comprehensive introduction to solid-state NMR spectroscopy for the analysis of polymer and hybrid electrolytes. Therefore, a table describing the necessary equipment to perform each experiment and the approximate time required is provided here (Table S1). NMR experimental techniques are listed in the order that they appear in the main text.

Table S1: Requirements for the analysis of solid polymer and hybrid electrolytes using the NMR spectroscopy experiments that are presented in the text

| NMR Experimental Technique | Required Equipment | Pulse Sequence | Experimental Time |
|---|---|---|---|
| PFG NMR | -High field magnet<br>-Multiple resonance gradient probe or MAS probe<br>-NMR tube or rotor<br>-Variable temperature capabilities (heater, chiller, heat exchanger) | Stimulated echo | Several Minutes |
| Electrophoretic NMR | - High field magnet<br>- Multiple resonance gradient probe<br>- Variable temperature capabilities (heater, chiller, heat exchanger)<br>- Specialized sample holder to supress convectional flow | Electrophoretic | Several Minutes |
| $T_1$ Relaxation | -High field magnet<br>-Magic angle spinning probe<br>-Rotor<br>-Variable temperature capabilities (heater, chiller, heat exchanger) | Inversion recovery | Minutes to hours depending on estimated $T_1$ relaxation time |
| $T_2$ Relaxation | -High field magnet<br>-Magic angle spinning probe<br>-Rotor | Carr-Purcell-Meilboom-Gill | Several minutes |

| | | | |
|---|---|---|---|
| | -Variable temperature capabilities (heater, chiller, heat exchanger) | | |
| Linewidth Analysis | -High field magnet<br>-Magic angle spinning probe<br>-Rotor<br>-Variable temperature capabilities (heater, chiller, heat exchanger) | Various1D experiments | Seconds to minutes depending on $T_1$ relaxation time |
| Exchange Spectrosocpy | -High field magnet<br>-Magic angle spinning probe<br>-Rotor<br>-Variable temperature capabilities (heater, chiller, heat exchanger) | EXSY | Several hours |
| Cross Polarization | -High field magnet<br>-Multi-resonance magic angle spinning probe<br>-Rotor<br>-Variable temperature capabilities (heater, chiller, heat exchanger) | Cross polarization | Seconds to minutes depending on $T_1$ relaxation time |
| REDOR | -High field magnet<br>-Magic angle spinning probe<br>-Rotor<br>-Variable temperature capabilities (heater, chiller, heat exchanger) | REDOR | Several minutes |
